# Machine-Learning Interatomic Potentials Achieving CCSD(T) Accuracy for Systems with Extended Covalent Networks and van der Waals Interactions

**DOI:** 10.1021/acs.jctc.5c02045

**Published:** 2026-03-03

**Authors:** Yuji Ikeda, Axel Forslund, Pranav Kumar, Yongliang Ou, Jong Hyun Jung, Andreas Köhn, Blazej Grabowski

**Affiliations:** † Institute for Materials Science, University of Stuttgart, Pfaffenwaldring 55, 70569 Stuttgart, Germany; ‡ Department of Materials Science and Engineering, KTH Royal Institute of Technology, SE-100 44 Stockholm, Sweden; § Department of Materials Science and Engineering, Massachusetts Institute of Technology, 77 Massachusetts Avenue, Cambridge, Massachusetts 02139, United States; ∥ Institute for Theoretical Chemistry, University of Stuttgart, Pfaffenwaldring 55, 70569 Stuttgart, Germany

## Abstract

Machine-learning interatomic potentials (MLIPs) enable large-scale atomistic simulations at moderate computational cost while retaining *ab initio* accuracy. In recent years, MLIPs trained on coupled-cluster data—particularly CCSD­(T), which includes single, double, and perturbative triple excitations—have emerged as a promising route to achieve chemical accuracy (1 kcal/mol) beyond the limits of density functional theory (DFT) and to incorporate nonempirical van der Waals (vdW) interactions. Most existing approaches are, however, still not straightforwardly applicable for systems with extended covalent networks such as covalent organic frameworks (COFs) due to the limited availability of CCSD­(T) under periodic boundary conditions. Here we present a methodology to train MLIPs with CCSD­(T) accuracy for systems with extended covalent networks. The approach is based on the Δ-learning method with a dispersion-corrected tight-binding baseline and an MLIP trained on the differences of the target CCSD­(T) energies from the baseline. This Δ-learning strategy enables training on compact molecular fragments while preserving transferability toward the periodic systems. Dispersion interactions are accounted for by including vdW-bound multimers in the training set, and the combination with a vdW-aware tight-binding baseline allows the formally local MLIP to attain CCSD­(T)-level accuracy even for systems dominated by long-range vdW forces. The resulting potential yields root-mean-square energy errors below 0.4 meV/atom on both training and test sets and reproduces electronic total atomization energies, bond lengths, harmonic vibrational frequencies, and intermolecular interaction energies for benchmark molecular systems. We apply the method to a prototypical quasi-two-dimensional covalent organic framework (COF) composed of carbon and hydrogen. The COF structure, interlayer binding energies, and hydrogen absorption are analyzed at CCSD­(T) accuracy. Overall, the developed methodology opens a practical route to large-scale atomistic simulations for systems with extended covalent networks and vdW interactions with chemical accuracy.

## Graphical abstract

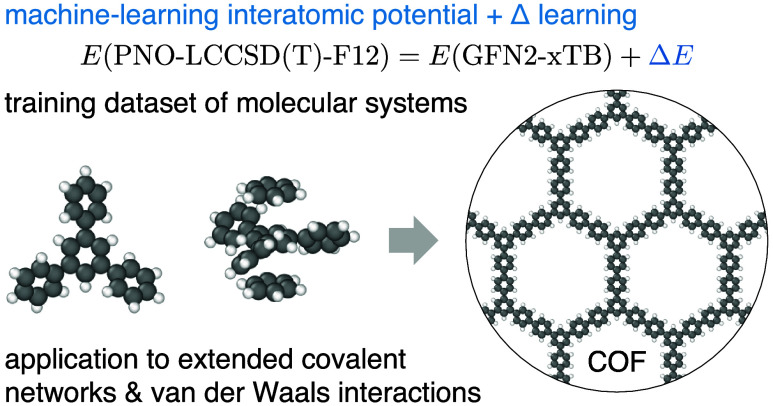

## I Introduction

Machine-learning interatomic potentials (MLIPs)^1^ are a new generation of interatomic potentials with flexible mathematics-oriented forms. MLIPs can accurately reproduce complicated potential-energy surfaces (PESs) of *ab initio* calculations, typically with errors below 1 meV/atom^2^ and at a fraction of the computational cost.

Most MLIPs to date are trained on density-functional theory (DFT) data. However, DFT suffers from errors due to the intrinsic approximations in exchange–correlation functionals and therefore often fails to reproduce experimental or higher-accuracy computational results.^3^ Moreover, the local density approximation (LDA) and the generalized gradient approximation (GGA) themselves cannot capture long-range van der Waals (vdW) interactions even qualitatively due to their local or semilocal nature. It is therefore common to add vdW interactions explicitly on top of these standard exchange–correlation functionals. These vdW functionals can be divided into element-specific schemes (e.g., the D4 correction^4,5^ ) and element-independent ones (e.g., the rVV10 correction^6–8^ ). However, both categories remain essentially semiempirical, as their parameters are tuned for limited benchmark sets, which can restrict their transferability.

Wave function-based post-Hartree–Fock (HF) methods^9,10^ can overcome the issues associated with DFT, because their accuracy can be improved systematically in a nonempirical manner and vdW interactions are intrinsically included. Particularly, the coupled-cluster method with single, double, and perturbative triple excitations (CCSD­(T))^11^ can achieve the so-called chemical accuracy of 1 kcal/mol (≈  40 meV/system), even below typical experimental errors, and is thus regarded as the gold standard of computational chemistry. However, the computational cost of canonical CCSD­(T) scales steeply as $O\left(N^{7}\right)$ with the number of correlated orbitals *N*, restricting routine applications to systems containing only a few dozen atoms.

MLIPs offer a way to sidestep the drawbacks of both DFT and CCSD­(T). When trained on CCSD­(T) reference data, MLIPs inherit its chemical accuracy and naturally incorporate vdW interactions while retaining the near-linear scaling of classical force fields. Various CCSD­(T)-level MLIPs have already been reported.^12–31^ One popular line of research trains MLIPs on CCSD­(T) potentials for small molecules or molecular clusters such as protonated water clusters;^12–16,20–22^ another targets liquid water.^23–27,31^ For wider chemical coverage, Smith et al.^17,18^ developed the ANI-1ccx potential trained on 5 00 000 configurations of monomer molecules computed at the CCSD­(T) level.

Despite these advances, most current CCSD­(T)-level MLIPs still lack application to systems featuring extended covalent networks, such as polymers, metal–organic frameworks (MOFs), and covalent–organic frameworks (COFs). The primary challenge is the limited availability of CCSD­(T) under periodic boundary conditions. Although explicit periodic implementations have been reported in recent years,^28,32–37^ they are not in broad routine use. To the best of our knowledge, so far only Herzog et al.^28^ employed their own periodic CCSD­(T) implementation to train an MLIP for a zeolite. For systems like molecular crystals and liquid water, fragmentation strategies^31,38,39^ can circumvent explicit periodic CCSD­(T) calculations. However, these approaches are not straightforwardly applicable to genuinely periodic systems with extended covalent networks; a naive cutting of such systems introduces unpaired valence electrons, and thereby qualitatively different electronic structures.

In the present study, we develop a methodology to train MLIPs with CCSD­(T) accuracy for systems with extended covalent networks and vdW interactions. Our methodology is based on the Δ-learning method with a dispersion-corrected tight-binding baseline and an MLIP trained on the differences of the target CCSD­(T) energies from the baseline. This strategy enables the MLIP to be trained solely on molecular systems while maintaining transferability to bulk materials. We validate the potential by comparing electronic total atomization energies (eTAEs), bond lengths, vibrational frequencies, and intermolecular interaction energies with high-level reference data, and then apply it to a prototypical COF to analyze its structure, interlayer binding energies, and hydrogen absorption at CCSD­(T) fidelity.

## II Methodology

### II.A Quantum Chemical Calculations

Quantum-chemical calculations for the training data set of the MLIP were performed with the MOLPRO 2024.1 program package.^40,41^ The energies were computed by the CCSD­(T) method augmented with F12 explicit correlation^42–44^ and employing the pair natural orbital (PNO) based local approximation, i.e., PNO-LCCSD­(T)-F12.^45,46^ The correlation-consistent polarized valence triple-ζ basis sets of Dunning^47^ augmented with diffuse functions^48^ for non-hydrogen atoms, i.e., heavy-aug-cc-pVTZ,^49,50^ were considered in the present study. Further details and the rationale behind the applied approaches are described in the following.

The computational cost of the CCSD­(T) method in the traditional canonical form scales as $O\left(N^{7}\right)$ , where *N* is the number of orbitals.^11^ This steep scaling of the computational cost limits the application of CCSD­(T) to only small molecules, particularly when a large basis set is used. Local approximations, particularly those based on PNOs,^45,46,51–68^ dramatically reduce the computational cost and can achieve near-linear scaling. This enables the computation for systems with a few hundreds of atoms at the CCSD­(T) level.

For the F12 correction, which dramatically reduces the basis-set-incompleteness error of the correlation energy,^44^ the F12b approximation^69^ with the 3*A ansatz^70,71^ and the diagonal fixed amplitude ansatz^42,43^ were employed.

The many-electron integrals in the F12 method were approximated by introducing the resolution of the identity (RI) with utilizing the complementary auxiliary basis set (CABS).^72^ The CABS singles correction^69,73^ was considered to reduce the basis-set-incompleteness error of the Hartree–Fock energy. All electrons including those in the core–shells were considered in the correlation methods and the CABS singles correction unless otherwise noted. The all-electron treatment substantially affects the eTAEs and the vibrational frequencies (Section III), while the impact on dispersion interaction can be marginal (Sec. S1 in the Supporting Information (SI)).

The basis-set-incompleteness error is typically mitigated by estimating the complete-basis-set (CBS) limit through an extrapolation combining two or more consecutive basis sets from a correlation-consistent family.^74,75^ However, for local-correlation methods, Werner and Hansen^76^ demonstrated that the basis-set convergence can become nonmonotonic because the local approximations introduce basis-set-dependent errors, and they instead recommended using the F12 correction. Guided by this finding, in the present study, we eschewed the basis-set extrapolation and employed the F12-corrected values obtained with the heavy-aug-cc-pVTZ basis set.

The basis-set-superposition error (BSSE), i.e., the error due to the basis-set-size difference among compared systems, is a particular type of the basis-set-incompleteness error. The BSSE can be particularly substantial for intermolecular interaction energies. Specifically, the total energy of a dimer system is not only modified by the interaction of the fragments but also by an improved description of the wave function of each fragment due to additional basis functions stemming from the other fragment. In general, the BSSE may be addressed using, e.g., the counterpoise (CP) correction.^77^ However, as written above, the F12 method dramatically reduces the basis-set-incompleteness error, which consequently also reduces the BSSE strongly.^78^ Combined with local-orbital methods, the BSSE is further suppressed.^79–82^ These BSSE reductions due to the F12 and the local-orbital approaches are also confirmed in the present study for a benzene–benzene dimer with π–π stacking as detailed in Sec. S1 in the SI. In short, in the PNO-LCCSD­(T)-F12 method without the CP correction, the errors from the reference data^83^ are less than 0.6 kcal/mol, which is smaller than the chemical-accuracy criterion of 1 kcal/mol. Therefore, in the present study, we did not apply the CP correction and used the raw PNO-LCCSD­(T)-F12 values for the sake of simplicity and reduction of computational cost.

The density-fitting approximation was applied to all the methods using the appropriate auxiliary basis sets for approximating Coulomb and exchange contributions to Fock matrix elements^84^ and two-electron integrals required in the correlation methods.^85^

For eTAEs, the spin-restricted open-shell variant of PNO-LCCSD­(T)-F12, i.e., PNO-RCCSD­(T)-F12 ^46^ was employed to compute the reference free-atom energies. The ^2^S state for H and the ^3^P state for C were considered. Note that the H atom is a single-electron system and the Hartree–Fock method already provides the corresponding energy.

For comparison, quantum-chemical calculations were also performed with other methods. These include the GGA in the Perdew–Burke–Ernzerhof (PBE) form^86^ without and with the D4 vdW correction,^4,87,88^ the second-order Møller–Plesset perturbation theory (MP2), CCSD, and CCSD­(T) in the non-PNO form without and with the F12 correction, and the random-phase approximation (RPA) based on PBE Kohn–Sham orbitals. The heavy-aug-cc-pVTZ basis set was used for these quantum-chemical calculations.

### II.B MLIP

#### II.B.1 Formalism

For the MLIP, we utilized the formalism of the moment-tensor potential (MTP).^89^ In the MTP formalism, the energy *E* ^MTP^ is the sum of contributions $V^{MTP}\left(n_{i}\right)$ of atomic neighborhoods $n_{i}$ for *N* atoms;

1 $$E^{MTP}=\overset{N}{\underset{i=1}{\sum }}V^{MTP}\left(n_{i}\right)$$

The neighborhood $n_{i}$ is represented as a tuple

2 $$n_{i}=\left(\left\{r_{i1},z_{i},z_{1}\right\},...,\left\{r_{ij},z_{i},z_{j}\right\},...,\left\{r_{iN_{nbh}},z_{i},z_{N_{nbh}}\right\}\right)$$

where *r*_*ij*_ are relative atomic positions, *z*_*i*_ and *z*_*j*_ are the types of central and neighboring atoms, and *N*_nbh_ is the number of atoms in neighborhood. Each contribution $V^{MTP}\left(n_{i}\right)$ in the potential energy *E* ^MTP^ expands through a set of MTP basis functions *B*_α_ as

3 $$V^{MTP}\left(n_{i}\right)=V_{0}\left(z_{i}\right)+\overset{N_{lin}}{\underset{\alpha =1}{\sum }}\xi_{\alpha }B_{\alpha }\left(n_{i}\right)$$

where ξ_α_ are the linear parameters to be optimized, and *N*_lin_ is the number of these parameters. *V*_0_ is the reference free-atom energy. To define the functional form of the MTP basis functions and the number *N*_lin_, the so-called moment-tensor descriptors are introduced;

4 $$M_{\mu ,\nu }\left(n_{i}\right)=\overset{N_{nbh}}{\underset{j=1}{\sum }}f_{\mu }\left(|r_{ij}|,z_{i},z_{j}\right)r_{ij}^{⊗\mu }$$

The angular part is given as the outer product of *r*_*ij*_ as

5 $$r_{ij}^{⊗\nu }\equiv \underset{\nu \mathrm{times}}{\underset{︸}{r_{ij}⊗\cdot \cdot \cdot ⊗r_{ij}\;}}$$

and thus a ν-th-order tensor. The radial part *f*_μ_(|*r*_*ij*_|, *z*_*i*_, *z*_*j*_) is a polynomial function smoothly truncated at the cutoff radius *R*_cut_. The MTP basis function *B*_α_ is a scalar value obtained by tensor contraction of one or more moment-tensor descriptors *M*_μ,ν_. The number of MTP basis functions is limited by the so-called MTP level, le*v*_max_. Specifically, the level of an MTP basis function *B*_α_ is defined as

6 $$lev\;B_{\alpha }\equiv \overset{P}{\underset{p=1}{\sum }}lev\;M_{\mu_{p},\nu_{p}}$$

where $M_{\mu_{p},\nu_{p}}$ are the moment-tensor descriptors involved in *B*_α_, and

7 $$lev\;M_{\mu ,\nu }\equiv 2+4\mu +\nu$$

For a given le*v*_max_, only the MTP basis functions with lev *B*_α_ ≤ le*v*_max_ are considered. Further details of the MTP formalism can be found in the literature.^89–91^

The training and evaluation of MTPs were performed using our own implementation,^92^ which allows fixing the reference free-atom energies *V*_0_ to those obtained in the quantum-chemical calculations. The MTP parameters were optimized in the procedure detailed in Sec. S4 in the SI, where the main optimization method was the Broyden–Fletcher–Goldfarb–Shanno (BFGS) algorithm as implemented in SciPy^93^ with a maximum number of 1000 iterations. The loss function optimized was the energy per atom averaged over the configurations in the training data set. The cutoff radius *R*_cut_ was set to 7 Å.

#### II.B.2 Δ-Learning

The major challenge for MLIPs with the CCSD­(T)-level accuracy^12–31^ is the high computational cost of CCSD­(T) to prepare sufficiently large training sets. The Δ-learning method^94,95^ is one of the popular approaches that can circumvent this issue and was employed in several previous studies.^31,96–102^ For example, Ruth et al.^99,100^ employed the Δ-learning method to predict the energies of small organic molecular systems in their ground-state structures at the CCSD­(T) level with the baseline of lower-accuracy methods including DFT. O’Neill et al.^31^ recently applied the Δ-learning method to develop CCSD­(T)-accuracy MLIPs for liquid water with the DFT baseline, which are available for molecular dynamics (MD) simulations at finite temperatures.

In the present study, the Δ-learning method is applied with the baseline of the tight-binding method, specifically the GFN2-xTB method.^103^ The energies of the molecular configurations in the training data set are thus decomposed as

E(PNO‐LCCSD(T)‐F12)=E(GFN2‐xTB)+ΔE 8

where *E*(PNO-LCCSD­(T)-F12) and *E*(GFN2-xTB) are the energies with the PNO-LCCSD­(T)-F12 and the GFN2-xTB methods, respectively, and Δ*E* is the energy difference between the two methods. An MTP is fitted to Δ*E* based on the energies of the molecular systems in the training data set. By summing up the energies or the forces of GFN2-xTB and the trained MTP, we reproduce the potential energy surface (PES) of the PNO-LCCSD­(T)-F12 method. We hereafter refer to the MTP trained on the Δ*E* as the ΔMTP and the sum of GFN2-xTB and ΔMTP as TB+ΔMTP.

In the above Δ-learning approach, it is supposed that major features of the PES are already well reproduced in GFN2-xTB and that Δ*E* provides corrections. A smaller number of configurations are therefore required to train an MTP for Δ*E* than for *E*(PNO-LCCSD­(T)-F12) directly. It is also assumed that the energetic differences between GFN2-xTB and CCSD­(T) are predominantly local. This allows us to train the ΔMTP that is available even for periodic systems such as COFs, where CCSD­(T) is not yet routinely available. In other words, the training data set for Δ*E* can still be constructed from the molecular systems that represent local regions of the target periodic systems that have extended covalent networks. Also, since GFN2-xTB is several orders faster than DFT, and an MLIP is even faster than GFN2-xTB, the Δ-learning approach makes it possible to evaluate the energies of the target system essentially only with the tight-binding cost. Another advantage of taking GFN2-xTB as the reference is that London dispersion interactions are already approximated with good accuracy by the D4 formalism.^4^ As demonstrated below for a benzene–benzene dimer with π–π stacking (Section III E), the London dispersion interaction at large distances is well reproduced already in GFN2-xTB, and therefore only the region around the equilibrium distance needs to be corrected. Therefore, the ΔMTP needs to be trained only with a moderate cutoff radius, which can also reduce the sizes of the molecular systems in the training data set.

The GFN2-xTB calculations were performed with the TBLITE code^104^ in combination with the interface for the ASE code.^105^ For the calculations of free atoms, an extension for open-shell high-spin states, i.e., spGFN2-xTB^106^ was employed.

#### II.B.3 Training, Validation, and Test Data Sets

COFs^108^ are nanoporous crystalline materials formed by covalent bonds of organic secondary building units composed mainly of light elements like C, N, O, as well as H. The covalent-bond networks are extended either in a two-dimensional (2D) or a three-dimensional (3D) way. The majority of experimentally synthesized COFs are quasi-2D layered materials^109–111^ held together by vdW interactions. Due to their lightweight, high porosity, and chemical diversity, COFs have attracted great interest for various applications, such as the storage and separation of gases like hydrogen (H_2_), methane (CH_4_), carbon dioxide (CO_2_), and ammonia (NH_3_),^112–114^ water harvesting,^115–117^ and photocatalysis for hydrogen evolution from water.^118–120^

To demonstrate the applicability of the developed Δ-learning approach, we considered a prototypical COF consisting of carbon and hydrogen synthesized in experiments on the Au(111) surface.^121,122^ This COF has a quasi-2D structure with each layer being a network of benzene rings. The 2D unit cell of a single layer is hexagonal, and the composition of each layer in the unit cell is C_48_H_30_. We therefore hereafter refer to this COF as the C_48_H_30_ COF. This COF is labeled 10020N2 or 16470N2 (both having the topologically identical structure) in the CURATED-COFs database.^111^ Figure 1(a) presents a single layer of the C_48_H_30_ COF.

**Figure 1:**
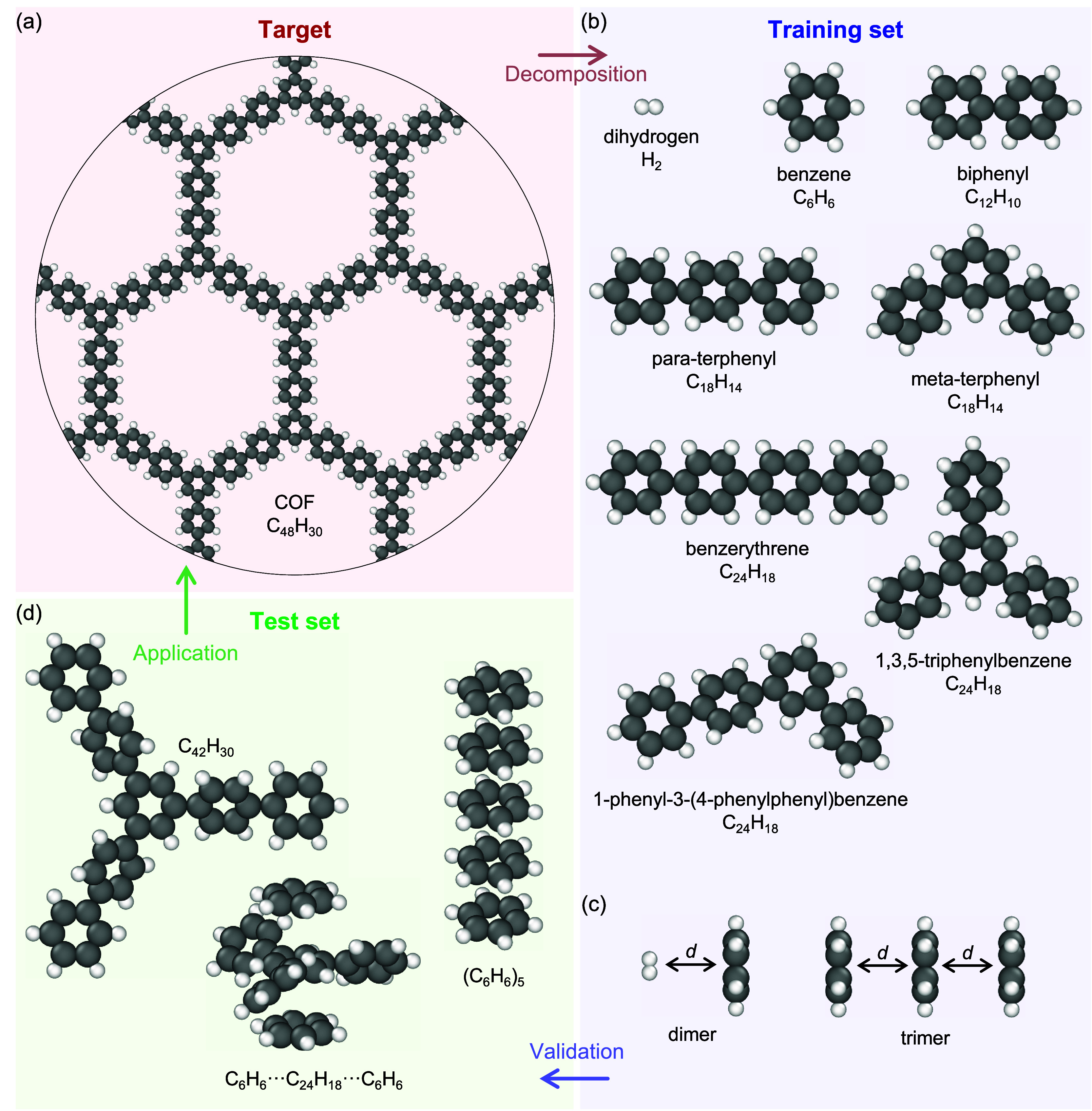
(a) Single layer of the periodic C_48_H_30_ COF. (b) Monomer molecules considered for the training of MTPs. (c) Dihydrogen–benzene dimer and benzene trimer with the center-of-mass distances *d* as examples of multimers considered also for the training. (d) Molecular systems used for testing the trained MTPs, thus not included in the training data sets. Visualization was performed using OVITO.^107^

The molecular systems for the training of the MTPs were selected from “components” of the target periodic COF, i.e., those that can be made by decomposing the COF into small fragments, with hydrogen termination to avoid unpaired valence electrons. Figure 1(b) shows the thus considered monomer molecules. In order to study also hydrogen absorption, dihydrogen (H_2_) was also included. To capture the interlayer vdW interaction, several dimers, trimers, and tetramers composed of these monomers were also included in the training set, as exemplified in Figure 1(c). In order to cover a wide range of intermolecular distance on an equal footing, ten center-of-mass distances between each monomer were considered. For each molecular system, MD simulations (detailed below) were conducted, and ten snapshots in the MD trajectory were selected. Nine among them were cumulated into the master training data set, while the last one was reserved for the validation of the MTPs after the training, which we hereafter refer to as the validation data set. Table 1 summarizes the considered molecular systems together with the numbers of configurations in both the data sets.

**Table 1:** Configurations in the Training, Validation, and Test Data Sets^a^

	System	*N*_ring_	*N*_isomer_	*N*_dist_	$N_{conf}^{train}$	$N_{conf}^{valid}$	$N_{conf}^{test}$
Monomer	H_2_	0	1	n/a	9	1	0
	C_6_H_6_	1	1	n/a	9	1	0
	C_12_H_10_	2	1	n/a	9	1	0
	C_18_H_14_	3	2	n/a	18	2	0
	C_24_H_18_	4	3	n/a	27	3	0
	C_42_H_30_	7	1	n/a	0	0	11
Dimer	H_2_···H_2_	0	1	10	90	10	0
	H_2_···C_6_H_6_	1	1	10	90	10	0
	H_2_···C_12_H_10_	2	1	10	90	10	0
	H_2_···C_18_H_14_	3	2	10	180	20	0
	C_6_H_6_···C_6_H_6_	2	1	10	90	10	0
	C_6_H_6_···C_12_H_10_	3	1	10	90	10	0
	C_6_H_6_···C_18_H_14_	4	2	10	180	20	0
	C_6_H_6_···C_24_H_18_	5	3	10	270	30	0
	C_12_H_10_···C_12_H_10_	4	1	10	90	10	0
Trimer	H_2_···H_2_···H_2_	0	1	10	90	10	0
	H_2_···6H_6_···H_2_	1	1	10	90	10	0
	H_2_···C_12_H_10_···H_2_	2	1	10	90	10	0
	C_6_H_6_···H_2_···C_6_H_6_	2	1	10	90	10	0
	C_6_H_6_···C_6_H_6_···C_6_H_6_	3	1	10	90	10	0
	C_6_H_6_···C_12_H_10_···C_6_H_6_	4	1	10	90	10	0
	C_6_H_6_···C_24_H_18_···C_6_H_6_	6	1	8	0	0	88
Tetramer	C_6_H_6_···C_6_H_6_···C_6_H_6_···C_6_H_6_	4	1	10	90	10	0
Pentamer	C_6_H_6_···C_6_H_6_···C_6_H_6_···C_6_H_6_···C_6_H_6_	5	1	8	0	0	88

a *N*_ring_: Number of benzene rings per isomer. *N*_isomer_: Number of isomers considered. *N*_dist_: Number of considered intermolecular distances. $N_{conf}^{train}$ : Number of configurations in the training data set. $N_{conf}^{valid}$ : Number of configurations in the validation set. $N_{conf}^{test}$ : Number of configuration in the test data set.

To assess the effect of the system size in the training data set on MTP quality, four training sets were constructed from the master training data set above based on the maximum number of benzene rings permitted in the systems. The first set included only molecules with up to two benzene rings, and the resulting ΔMTP is labeled #2. Analogously, three more training sets allowed up to three, four, and five benzene rings, yielding models #3, #4, and #5, respectively. Specifically, ΔMTP#5 included 8 monomers, 12 dimers, 6 trimers, and 1 tetramer, containing at maximum 30 carbon atoms.

In addition to the molecular systems in the training data sets, we also evaluated larger molecular systems as test data sets to examine whether the trained MTPs retain predictive accuracy for systems beyond those used in training, thereby indirectly supporting their transferability to the periodic C_48_H_30_ COF. The systems for the test data set consist of a 1,3,5-tri­(4-biphenyl)­benzene monomer (C_42_H_30_), a benzene–1,3,5-triphenylbenzene–benzene trimer (C_6_H_6_···C_24_H_18_···C_6_H_6_), and a benzene pentamer ((C_6_H_6_)_5_), as presented in Figure 1(d).

The data sets of MLIPs for finite-temperature simulations are often made by MD simulations,^2,123–141^ the strategy adopted also in the present study. As MD simulations are prohibitively expensive at the coupled-cluster level, we instead used GFN2-xTB for MD simulations. For selected snapshots from these trajectories, PNO-LCCSD­(T)-F12 single-point energies were computed.

Further details of the geometry generation are as follows. The geometries of the monomers were first obtained from the ChemSpider Web site.^142^ They were then relaxed with the GFN2-xTB method using the BFGS algorithm implemented in ASE^105^ until all the forces on atoms became less than 0.001 eV/Å. The MD simulations were then performed starting from the relaxed geometries in the Langevin thermostat implemented in ASE at 300 K with a friction parameter of 0.01 fs^–1^ for a simulation time of 1 ps with a time step of 1 fs, thus 1001 steps including the zeroth step. For multimers, their constituent monomers were initially aligned along the axis corresponding to the largest principal moment of inertia, and the center-of-mass distances of the neighboring monomers were fixed to a given value during the MD simulations. Ten values in 3.0–7.5 Å with a step of 0.5 Å were considered as the center-of-mass distances for the training and the validation data sets, while eight values in 4.0–7.5 Å were considered for the test data set. (Shorter distances were excluded because quantum-chemical calculations often raised errors due to basis-set linear dependence.) Thus, specifically for ΔMTP#5, 1872 and 208, and 187 configurations were in the training, validation, and test data sets.

#### II.B.4 Local Extrapolation Grade

The trained ΔMTPs were also evaluated based on their uncertainties for given configurations. Specifically, we employed the extrapolation grade^143,144^ defined based on D-optimality. The extrapolation grade evaluates the similarity of the given configurations or the local atomic environments to those in the training data set. A value less than 1 indicates that the considered configuration of the local environment is interpolative and that the prediction should be reliable, while a higher extrapolation grade implies a larger uncertainty for properties predicted by the potential. The extrapolation grade is advantageous because this can be evaluated only based on geometry and thus for periodic systems. The extrapolation grade has often been employed as the uncertainty measure during active learning.^124,134,145,146^ As applied for high-entropy alloys in our previous study,^124^ an extrapolation grade less than 2 should be safe for application.

The extrapolation grade can be defined both for a whole configuration and for each atom,^143,144^ and in the present study, we considered the latter one, which we hereafter refer to as the local extrapolation grade. The MLIP3 package^91^ was employed for the calculation of the local extrapolation grade. The local extrapolation grades were evaluated for the molecular systems in the test data set and for the periodic C_48_H_30_ COF.

#### II.B.5 Comparison with ANI-1ccx

The performance of TB+ΔMTP was compared with another MLIP, ANI-1ccx.^17,18^ The ANI-1ccx potential is an ANI-style neural network potential^147^ trained on the CCSD­(T)-level training data set aiming for broad organic chemistry applications. As many as 500000 configurations of molecules consisting of the elements in {H, C, N, O} were employed for the training, while H_2_ is absent in the data set. The CCSD­(T)-level calculations were performed with the cc-pV*X*Z basis sets up to with *X* in {D, T}, without augmenting diffuse functions. The cutoff radius was set to 5.2 Å. Free-atom energies were obtained by linear fitting to the energy per atomic species in the training data set. The ANI-1ccx calculations were performed with the TorchANI code^148^ with the interface for the ASE code.^105^

## III Results and Discussion

### III.A RMSEs of MTPs

Figure 2(a) shows the root-mean-square errors (RMSEs) of the energies per atom for the molecular systems in the training and the validation data sets predicted by ΔMTPs for several MTP levels. Table 2 summarizes the RMSEs for the data sets obtained with the ΔMTPs. For the training data sets, all the ΔMTPs show monotonous decreases of RMSEs with increasing MTP level, i.e., number of parameters. For the validation data set, in contrast, ΔMTP#2 shows about one order larger RMSEs than those for the training data set. This implies overfitting due to less configurations in the corresponding training data set with respect to the number of MTP parameters as well as due to the incapability to predict larger molecules in the validation data set not included in the training data set. With increasing the sizes of molecular systems in the training data set, the difference between the RMSEs for the training and the validation data sets becomes smaller, demonstrating the suppression of the overfitting above. Particularly for ΔMTP#4 and ΔMTP#5 at MTP levels of 16 and 20, the RMSEs for the validation data set are about 0.4 meV/atom or lower. For these ΔMTPs, however, while the RMSEs for the validation data set decrease monotonously until an MTP level of 16, the RMSEs of the MTP levels of 16 and 20 are almost the same. This implies that a further higher MTP level would not further improve the transferability of the MTP due to overfitting. The test data set also shows similar RMSEs as the validation data sets, as found in Figure 2(b), indicating that the predictive power is preserved even for the extended molecular systems not included in the training data sets.

**Figure 2:**
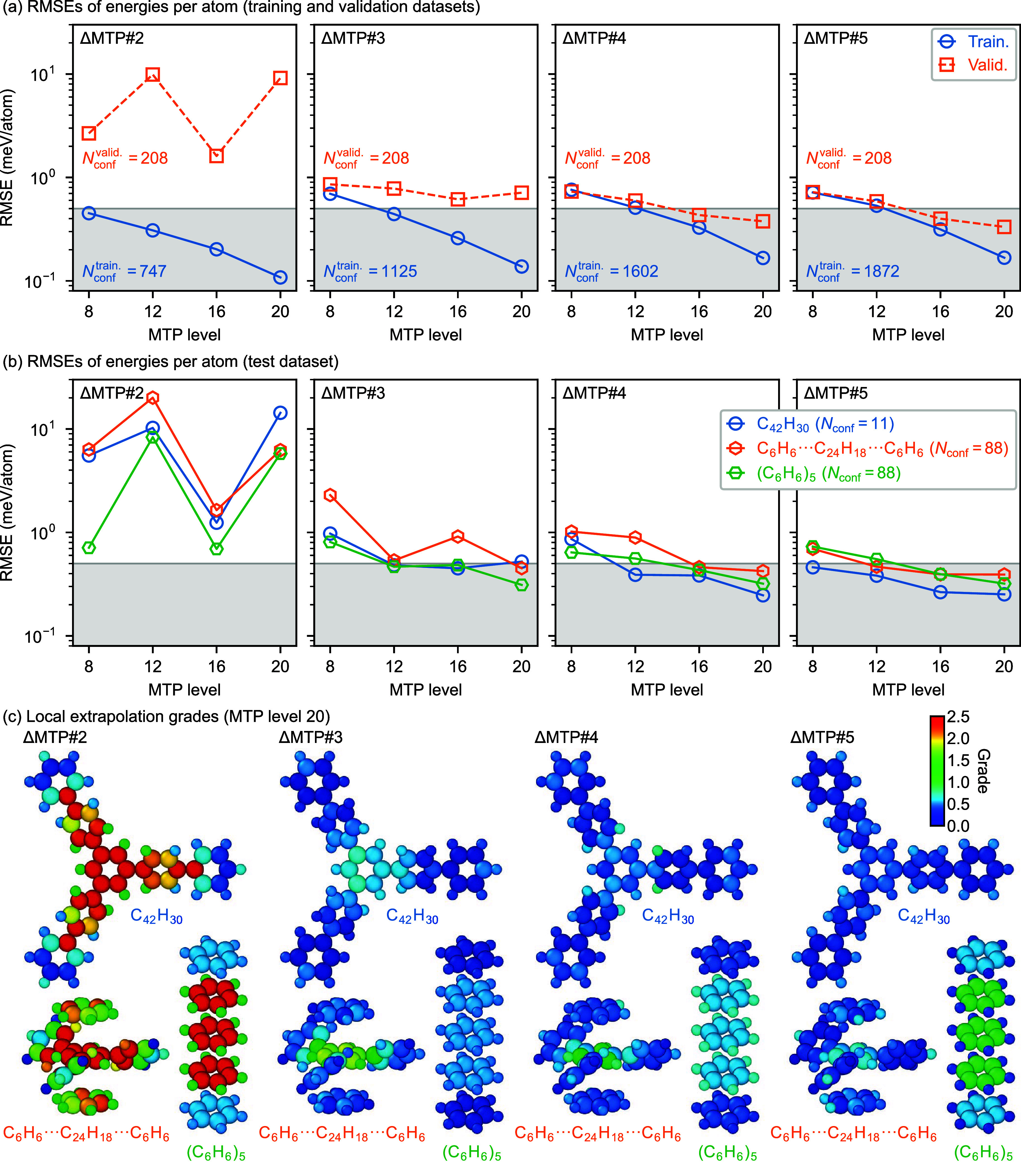
(a) RMSEs of the energies per atom for the molecular systems in the training and the validation data sets predicted by ΔMTPs. (b) RMSEs of the energies per atom for the molecular systems in the test data set. (c) Local extrapolation grades of a selected snapshot from each molecular system in the test data set obtained with ΔMTPs at level 20.

**Table 2:** RMSEs (in meV/atom) for the Investigated Data Sets Obtained with ΔMTP#*N*

*N*	Level	Training #*N*	Validation	Test
2	8	0.451	2.671	4.577
	12	0.308	9.888	15.119
	16	0.202	1.613	1.259
	20	0.108	9.147	6.797
3	8	0.695	0.857	1.687
	12	0.443	0.781	0.501
	16	0.259	0.615	0.717
	20	0.138	0.712	0.397
4	8	0.760	0.730	0.852
	12	0.510	0.598	0.729
	16	0.327	0.433	0.444
	20	0.166	0.377	0.368
5	8	0.715	0.721	0.700
	12	0.533	0.587	0.504
	16	0.315	0.399	0.387
	20	0.168	0.333	0.352

Figure 2(c) visualizes the local extrapolation grades of selected configurations in the test data set obtained with the ΔMTPs. The extrapolation grades tend to be lower for the atoms in the exterior region of each system, and those for the atoms in the interior region tend to be higher. Particularly for ΔMTP#2, the extrapolation grades in the interior regions are higher than 2.5 and thus indicate large uncertainty for this ΔMTP. In contrast, for ΔMTPs with larger training data sets, the extrapolation grades are much lower, supporting the transferability of these MTPs even for the extended systems not included in the training data sets. The lowering of the extrapolation grades are found not only for the monomer molecule (C_42_H_30_) but also for the benzene pentamer ((C_6_H_6_)_5_), meaning that the uncertainty decreases not only for covalent interactions but also for vdW interactions. This improvement demonstrates that the strategy to apply local MLIPs to extended systems should work in combination with the Δ-learning approach with the GFN2-xTB baseline.

Figure 3 shows the differences of the cohesive energies per atom for the #5 training data set computed with various methods with respect to the reference PNO-LCCSD­(T)-F12 values. The errors due to the MTP fitting are three orders smaller than the errors due to different methods, e.g., PBE-D4, indicating that TB+ΔMTP#5 predicts the CCSD­(T)-level energies much more accurately than the other methods.

**Figure 3:**
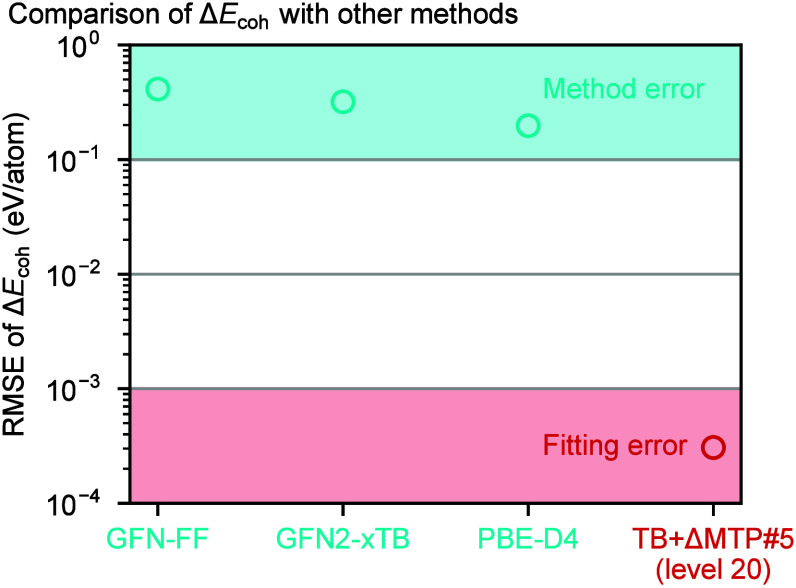
RMSEs of cohesive energies per atom for the training data sets in GFN-FF,^149^ GFN2-xTB,^103^ PBE-D4,^4,86^ as well as TB+ΔMTP#5 at the MTP level 20 with respect to the reference PNO-LCCSD­(T)-F12 values.

Unless otherwise stated, we hereafter focus on the results with TB+ΔMTP#5 at level 20.

### III.B Electronic Total Atomization Energies of H_2_ and C_6_H_6_

Table 3 shows the eTAEs of dihydrogen (H_2_) obtained by (sp)­GFN2-xTB, various quantum-chemical methods, ANI-1ccx, and TB+ΔMTP#5, as well as the experimental values (where the contributions of zero-point vibrations are subtracted).^150,151^ The GFN2-xTB method underestimates the TAEs of H_2_ by 31.7 kcal/mol. The errors are largely reduced in DFT. For PNO-LCCSD-F12, the deviation from the experimental reference is only 0.3 kcal/mol, well below the chemical-accuracy criterion of 1 kcal/mol. (Note that H_2_ is a two-electron system, and therefore there is no triples correction.) TB+ΔMTP#5 essentially perfectly reproduces the target PNO-LCCSD-F12 value. In contrast, ANI-1ccx shows a huge underestimation of the TAE, likely because its training data set does not include H_2_.

**Table 3:** Electronic Total Atomization Energy (kcal/mol) of H_2_ ^a^

Method	eTAE
GFN2-xTB	77.8
PBE	104.7
PBE-D4	104.8
PNO-LCCSD-F12^b^	109.2
ANI-1ccx^c^	1.0
**TB+ΔMTP#5**	109.2
Exp.^d^	109.5

a The heavy-aug-cc-pVTZ basis set was used for quantum-chemical calculations.

b H_2_ has only two electrons and therefore no triple excitations.

c Free-atom energies were obtained by linear fitting to the energy per atomic species in the training data set.

d Huber and Herzberg^150^ with subtracting the vibrational zero-point energy from Irikura.^151^

Table 4 shows the eTAEs of benzene (C_6_H_6_) computed in various approaches. The (sp)­GFN2-xTB method shows an overestimation by 57.8 kcal/mol from the experimental value.^152^ The errors are still large in the DFT methods; for example, PBE shows an error of 47.3 kcal/mol, far beyond the chemical accuracy. The D4 dispersion correction makes the deviation even larger.

**Table 4:** Electronic Total Atomization Energy (kcal/mol) of C_6_H_6_ ^a^

Method	Correlation	eTAE
GFN2-xTB	n/a	1425.6
PBE	n/a	1414.1
PBE-D4	n/a	1420.8
PNO-LCCSD(T)-F12	frozen-core	1360.9
	all-electron	1366.5
ANI-1ccx^b^	n/a	39.4
**TB+ΔMTP#5**	n/a	1366.5
Exp.^c^	n/a	1367.8(7)

a The heavy-aug-cc-pVTZ basis set was used for quantum-chemical calculations.

b Free-atom energies were obtained by linear fitting to the energy per atomic species in the training data set.

c Estimation of Parthiban and Martin, which intrinsically contains scalar relativistic effects (obtained as –0.99 kcal/mol) and spin–orbit coupling (−0.51 kcal/mol).^152^

The all-electron correlation treatment is found essential for accurate calculations of TAEs for PNO-LCCSD­(T)-F12. In the frozen-core approximation, PNO-LCCSD­(T)-F12 shows a deviation of –6.9 kcal/mol from experiments, while the error becomes much smaller in the all-electron treatment, –1.3 kcal/mol, near the chemical accuracy. A similar impact of all-electron treatment (7.1 kcal/mol) was found also in an accurate coupled-cluster level *ab initio* calculation by Parthiban and Martin.^152^

TB+ΔMTP#5 again essentially perfectly reproduces the target value of PNO-LCCSD­(T)-F12 and thus yields the same small deviation from experiments. In contrast, ANI-1ccx shows a large error of –1328.4 kcal/mol. This is likely because free-atom energies in ANI-1ccx are determined by linear fitting over the entire data set of molecules rather than by actual quantum-chemical calculations.

### III.C Bond Lengths of H_2_ and C_6_H_6_

Figure 4 presents the equilibrium bond lengths of dihydrogen (H_2_) obtained using GFN2-xTB, several quantum-chemical methods, ANI-1ccx, and TB+ΔMTP#5, as well as the value derived from experiments (without contributions of zero-point vibrations).^150^ The GFN2-xTB method leads to an overestimation of about 0.04 Å relative to the experimental value. The DFT methods show substantially smaller deviations by approximately 0.01 Å. Notably, the PBE-D4 vdW functional does not improve the bond length over the PBE result. The post-HF methods show even smaller deviations from experiments; particularly, PNO-LCCSD-F12 shows a deviation as small as 0.0001 Å, indicating the accuracy of the method. TB+ΔMTP#5 reproduces the target value of PNO-LCCSD-F12 almost perfectly, with a deviation of less than 0.000 01 Å. ANI-1ccx does not reproduce the H–H bond length even qualitatively, again because the training data set for this potential does not include H_2_.

**Figure 4:**
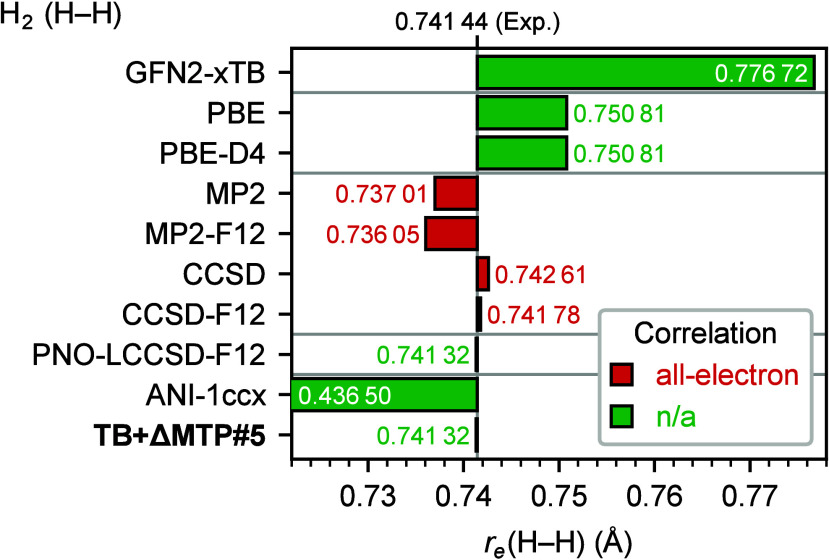
Equilibrium bond length (Å) of dihydrogen (H_2_) with respect to the experimental value in Huber and Herzberg.^150^ The heavy-aug-cc-pVTZ basis set was used for quantum-chemical calculations. Note that H_2_ has only two electrons and therefore no triple excitations. The PNO-LCCSD­(T)-F12 value was obtained by fitting the energies for H–H bond lengths on a 0.001 Å grid to a univariate second-order polynomial.

Figure 5 presents the equilibrium bond lengths of benzene (C_6_H_6_) obtained using GFN2-xTB, several quantum-chemical methods, ANI-1ccx, and TB+ΔMTP#5, as well as the values derived from experiments (without contributions of zero-point vibrations).^153,154^ The DFT values deviate from experiments by less than 0.006 Å for the C–C bonds but as large as 0.01 Å for the C–H bonds. PBE-D4 exhibits deviations virtually identical to those of plain PBE, as with H_2_ above.

Regarding post-HF methods, it is worth first discussing the relation between all-electron treatment and F12 explicit interelectronic correlation. Using the frozen-core approximation, MP2, CCSD, and CCSD­(T) overestimate C–C bond lengths by about 0.007 Å and C–H bond lengths by about 0.002 Å relative to all-electron values. Adding the F12 explicit-electron-correlation method reduces these errors to roughly 0.001–0.002 Å. Apparent discrepancies between frozen-core and all-electron treatments in non-F12 calculations likely arise from different rates of basis-set convergence for the two approaches; F12 accelerates this convergence and thus largely removes those discrepancies. In PNO-LCCSD­(T)-F12, the bond lengths are close to the experimental values with overestimation of as small as 0.001–0.003 Å.

TB+ΔMTP#5 shows an excellent agreement to the reference PNO-LCCSD­(T)-F12 with the all-electron treatment with errors about 0.0003 Å or less both for the C–C and the C–H bond lengths. Eventually, the errors from experimental values are less than 0.002 Å. ANI-1ccx shows a similar error for the C–C bond length but a larger error of 0.004 Å for the C–H bond length.

### III.D Vibrational Frequencies of H_2_ and C_6_H_6_

Figure 6 presents the RMSEs of harmonic vibrational frequencies computed with GFN2-xTB, various quantum-chemical methods, ANI-1ccx, and TB+ΔMTP#5 with respect to experimental values.^150,156,157^ Sec. S5 in the SI provides the full values of the obtained harmonic vibrational frequencies. The harmonic vibrational frequencies were computed with the finite-displacement method with a displacement of 0.01 Å and analytical forces using our own implementation. Note that the PNO methods currently have no implementation of analytical forces in MOLPRO and are therefore not considered in this part.

**Figure 5:**
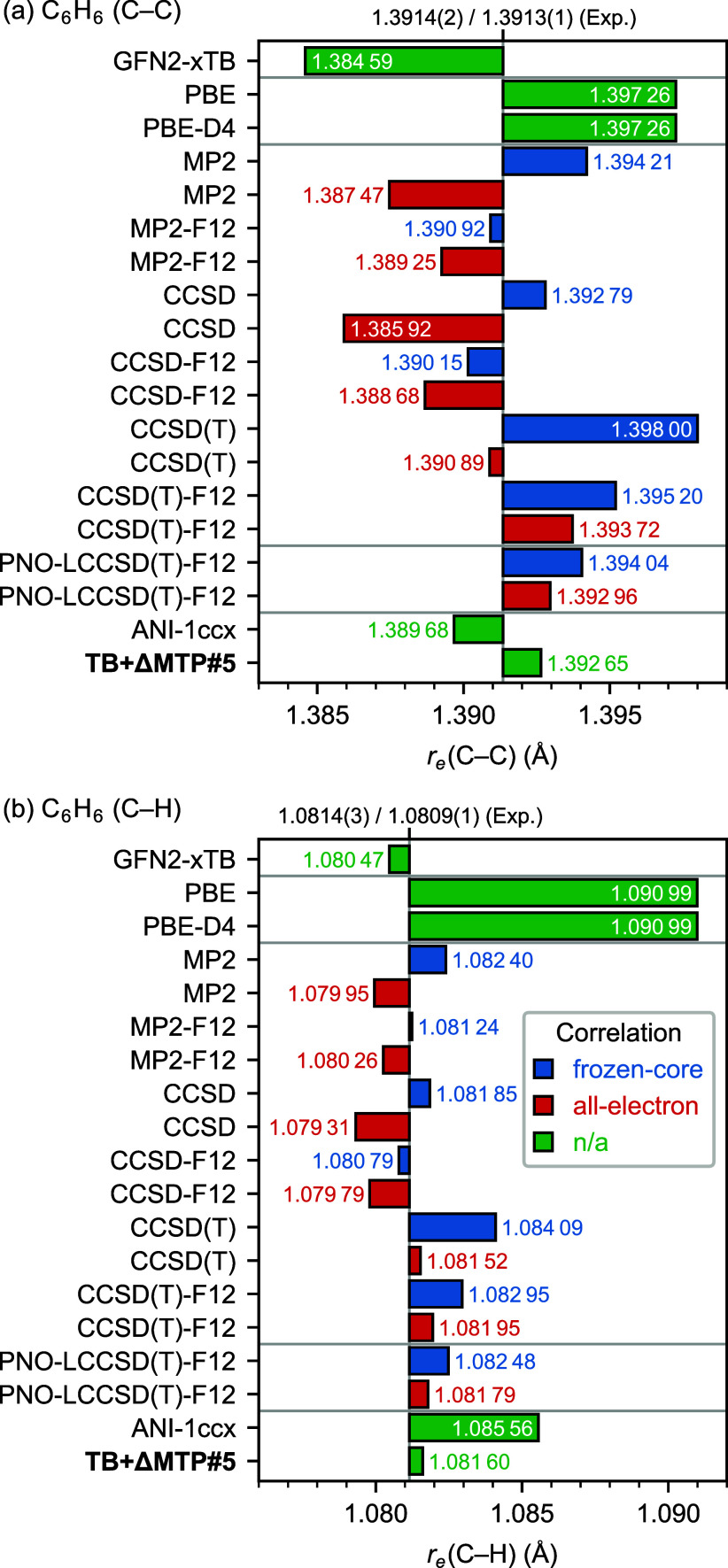
Equilibrium bond lengths (Å) of benzene (C_6_H_6_) with respect to the experimental values of Heo et al.^153^ and Esselman et al.^154^ The heavy-aug-cc-pVTZ basis set was used for quantum-chemical calculations. The PNO-LCCSD­(T)-F12 values were obtained by fitting the energies for (a) C–C and (b) C–H bond lengths on a 0.001 Å grid to a bivariate second-order polynomial.

**Figure 6:**
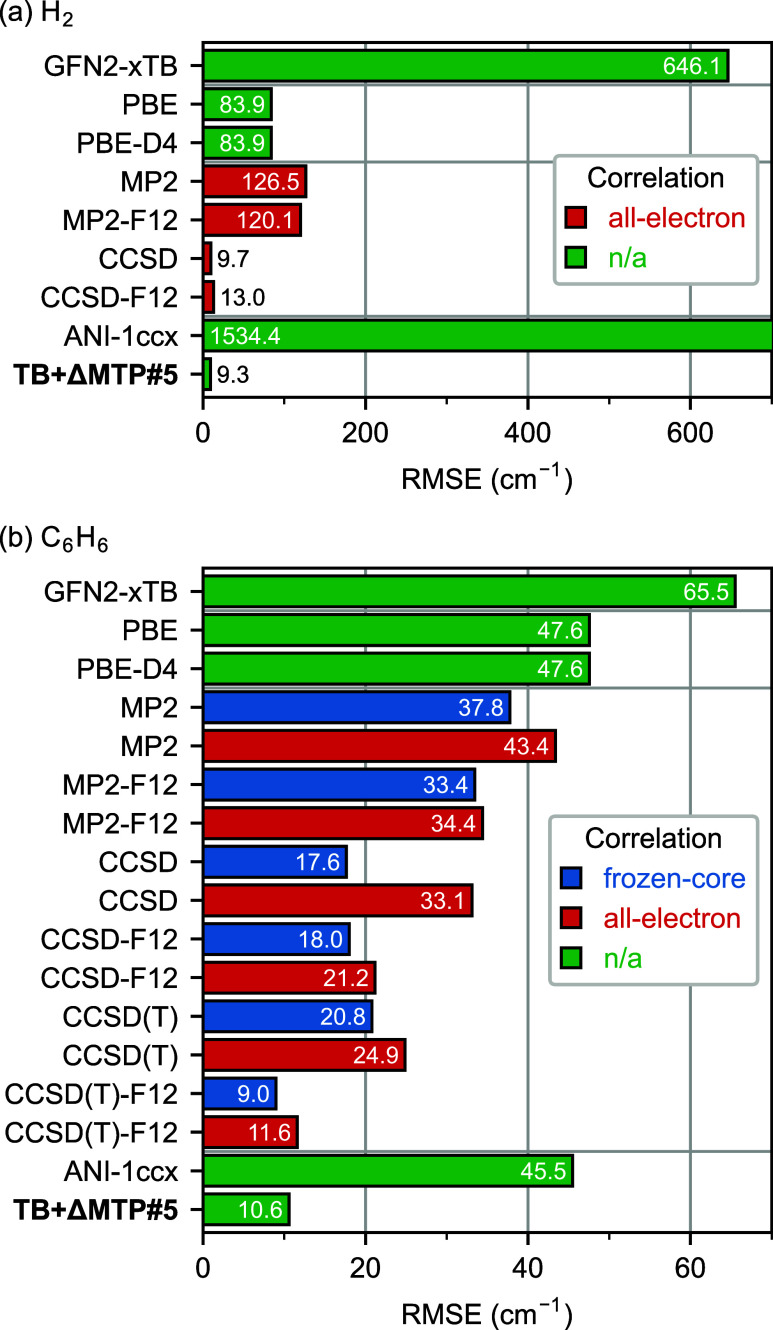
RMSEs of harmonic vibrational frequencies with respect to the experimental values. (a) H_2_. (b) C_6_H_6_. The heavy-aug-cc-pVTZ basis set was used for quantum-chemical calculations.

For H_2_ (Figure 6(a)), GFN2-xTB shows a large deviation of 646.1 cm^–1^. The error is largely reduced in DFT, particularly in PBE­(-D4) (83.9 cm^–1^). While MP2 and MP2-F12 show larger errors than PBE (126.5 cm^–1^ and 120.1 cm^–1^, respectively), the errors in CCSD and CCSD-F12 are as small as 9.7 cm^–1^ and 13.0 cm^–1^, respectively. TB+ΔMTP#5 also shows a small RMSE comparable with CCSD and CCSD-F12, 9.3 cm^–1^. In contrast, ANI-1ccx shows an error as large as 1534.4 cm^–1^, which is again due to the absence of H_2_ in the ANI-1ccx training data set.

For C_6_H_6_ (Figure 6(b)), GFN2-xTB shows an error of 65.5 cm^–1^, and DFT shows slightly smaller deviations (47.6–54.3 cm^–1^). For the post-HF methods, the all-electron treatment with the present heavy-aug-cc-pVTZ basis set leads to larger deviations from experiments than the frozen-core approximation. This impact is insignificant for the F12 methods but substantial for the non-F12 methods. CCSD­(T)-F12 with the frozen-core approximation and the all-electron treatment shows errors of 9.0 cm^–1^ and 11.6 cm^–1^, respectively. The error of TB+ΔMTP#5 is 10.6 cm^–1^, comparable with CCSD­(T)-F12. In contrast, the error of ANI-1ccx, 45.5 cm^–1^, is only comparable with the DFT and the MP2 methods.

The success of TB+ΔMTP#5 in reproducing the vibrational frequencies is likely due to the incorporation of MD snapshots into the training data set. Thus, we may conclude that TB+ΔMTP#5 has an accuracy beyond DFT, nearly reaching the CCSD­(T) level. Note that the D4 vdW correction on PBE does not improve the vibrational frequencies, like for bond lengths (Section III C), demonstrating again that such a semiempirical vdW-specific correction cannot fully replace quantum-chemical calculations.

### III.E Intermolecular Interaction Energies of C_6_H_6_

Figure 7 depicts the intermolecular interaction energy curves of a benzene–benzene dimer with π–π stacking computed with GFN2-xTB, various quantum-chemical methods, ANI-1ccx, and TB+ΔMTP#5. The geometries are taken from the S66 × 8 data set^155^ revised in ref^83^ alongside the reference values of the CCSD­(F12*)­(T) method^158^ at the CBS limit. For the quantum-chemical calculations, except PNO-LCCSD­(T)-F12, the counterpoise (CP) correction is applied. Below we first review the results of the quantum-chemical calculations and then discuss the quality of TB+ΔMTP#5.

**Figure 7:**
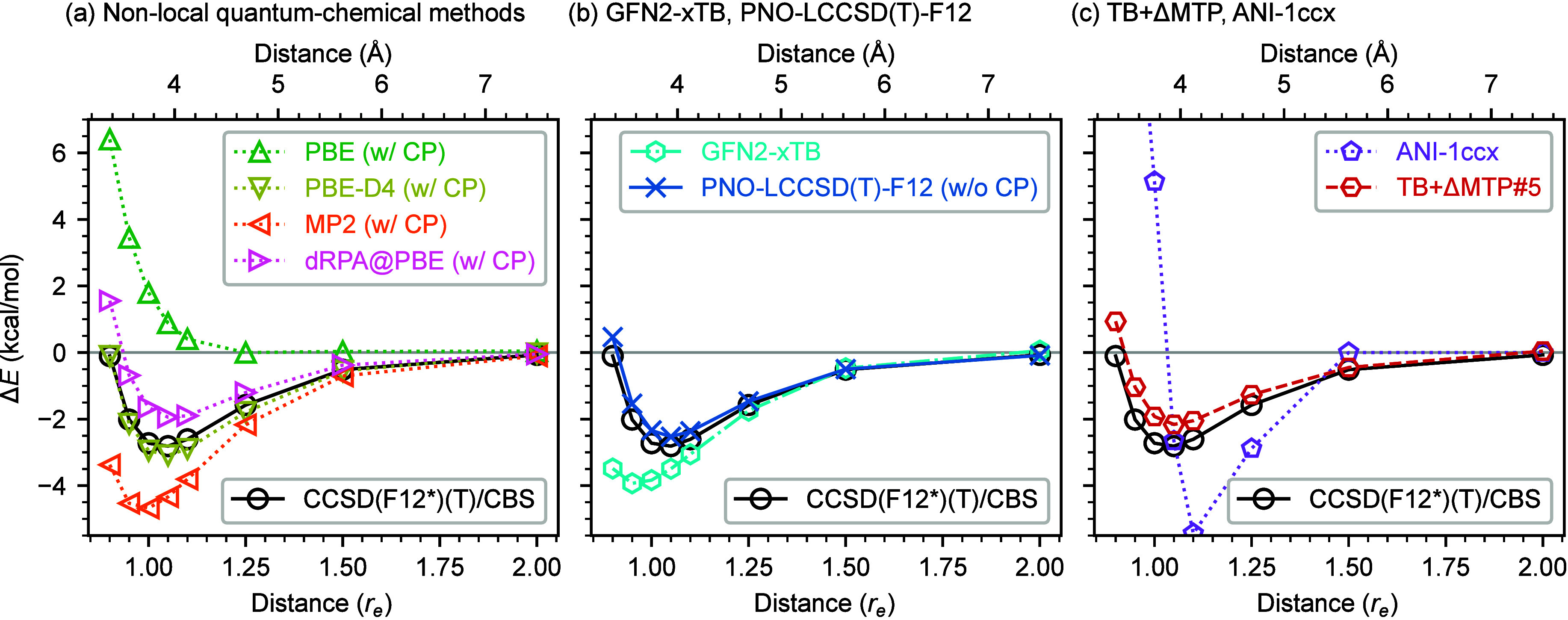
Intermolecular interaction energies of a benzene–benzene dimer with π–π stacking. The geometries refer to the S66 × 8 data set.^83,155^ The solid black curve shows the CCSD­(F12*)­(T)/CBS values from the S66 × 8 data set^83^ for reference. The *x*-axis shows the intermolecular distance scaled by the equilibrium value *r*_*e*_ provided in the S66 × 8 data set.

In Figure 7(a), PBE cannot predict the dimer attraction even qualitatively, as is well known, due to the absence of the vdW interaction in this exchange–correlation functional and consistent with a previous study.^159^ The agreement is largely improved in PBE-D4, as however the parameters in the D4 formalism are determined to reproduce the S66 × 8 values semiempirically.^4,87^ The dimer attraction is qualitatively reproduced in the MP2 method. However, the strength of the binding is largely overestimated, and the equilibrium dimer distance is substantially underestimated. The direct RPA (dRPA) method shows a much better agreement with the reference values compared to DFT and MP2. However, this agreement largely owes to the CP correction. As detailed in Sec. S3 in the SM, without the CP correction, the magnitude of the binding energy is overestimated, and particularly when an all-electron treatment is employed, the deviation is significant, meaning that the CP correction is essential if the RPA methods are used to generate a training data set for an MLIP. However, routine applications of the CP correction are highly cumbersome, particularly for systems with more than two molecules. In this regard, the RPA methods are not suitable for making an MLIP applicable particularly for systems with intermolecular interactions.

In Figure 7(b), PNO-LCCSD­(T)-F12 agrees well with the reference values^83^ of CCSD­(F12*)­(T)/CBS with errors less than 0.6 kcal/mol across all distances examined. Further, unlike the RPA methods above, PNO-LCCSD­(T)-F12 does not reveal a substantial BSSE (Sec. S1 in the SI) and thus does not require the CP correction. Hence, the calculations of multimolecule systems for the training of an MLIP can be performed on an equal footing as for multimers. Note that a recent study by Hansen et al.^160^ demonstrates that further refining of the domain and the pair approximations in PNO-LCCSD­(T)-F12 increases the magnitude of the equilibrium interaction energy by about 0.1 kcal/mol, thereby narrowing the gap with the reference CCSD­(T)/CBS value, though at the expense of longer computation times. The agreement of GFN2-xTB with the reference CCSD­(F12*)­(T)/CBS values^83^ is better than MP2. Since the computational cost of GFN2-xTB is much cheaper than DFT and post-HF, GFN2-xTB is a better baseline for the Δ-learning approach.

In Figure 7(c), TB+ΔMTP#5 well reproduces the PNO-LCCSD­(T)-F12 values and thus also the reference S66 × 8 values,^83^ with an error of less than 0.6 kcal/mol at *r*_e_ = 1.00. In contrast, ANI-1ccx cannot reproduce the reference energies even qualitatively. This is likely due to a bias in the ANI-1ccx data set. Specifically, while the ANI-1ccx data set^17,18^ includes as many as 500000 structures, most of them are monomers of relatively small molecules and thus do not include London dispersion interaction. Furthermore, the interaction energies predicted by ANI-1ccx fall off to zero for distances larger than 1.5*r*_*e*_ ≈ 5.6 Å (larger than the cutoff radius of ANI-1ccx) due to the absence of any long-range interactions.

### III.F Application to the C_48_H_30_ COF

Having thus verified the performance of TB+ΔMTP#5, we next apply this potential to the periodic C_48_H_30_ COF. For comparison, the results with GFN2-xTB and PBE-D4 are also shown. All calculations employed a six-layer simulation cell containing 468 atoms. For the tight-binding and the DFT calculations, the reciprocal spaces of the 468-atom simulation cells were sampled by the Γ-point-only mesh. Further details on the DFT calculations are provided in Sec. S6 in the SI.

We first considered fully eclipsed layer stacking with perfectly planar layers, as shown in Figure 8(a), which shows a space group type of *P*6/*mmm* (No. 191). However, its phonon density of states (DOS) (Figure 9(a)) shows numerous imaginary modes, indicating that this structure is dynamically unstable at 0 K. Therefore, to further relax the structure, we once broke its symmetry by perturbing the positions of the atoms by a Gaussian distribution with a standard variation of 0.01 Å and then rerelaxed the structure. After the relaxation and symmetry refinement, the structure shows a space group type of *C*222 (No. 21). In this structure, the layers are not fully planar anymore but rather show a twisting between in-plane adjacent benzene rings, as shown in Figure 8(b). The energy of the *C*222 structure is lower by 10.2 meV/atom than the *P*6/*mmm* structure, as given in Table 5. The phonon DOS of the *C*222 structure shows no imaginary modes (Figure 9(b)), supporting its dynamical stability. The same trend is obtained also for GFN2-xTB and the PBE-D4 vdW DFT functional. The twisting is well consistent with molecular systems like biphenyl.^161^

**Figure 8:**
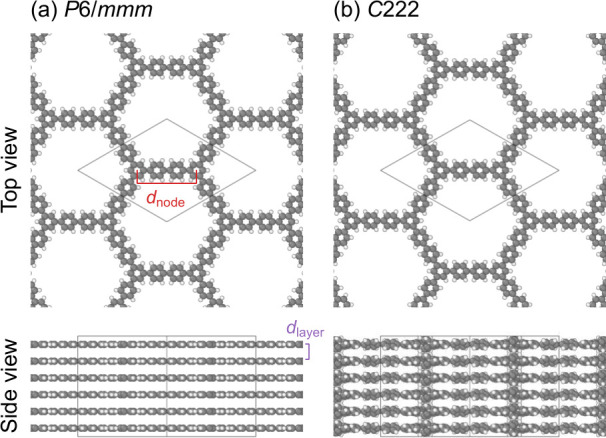
Structures of the C_48_H_30_ COF optimized with TB+ΔMTP#5.

**Figure 9:**
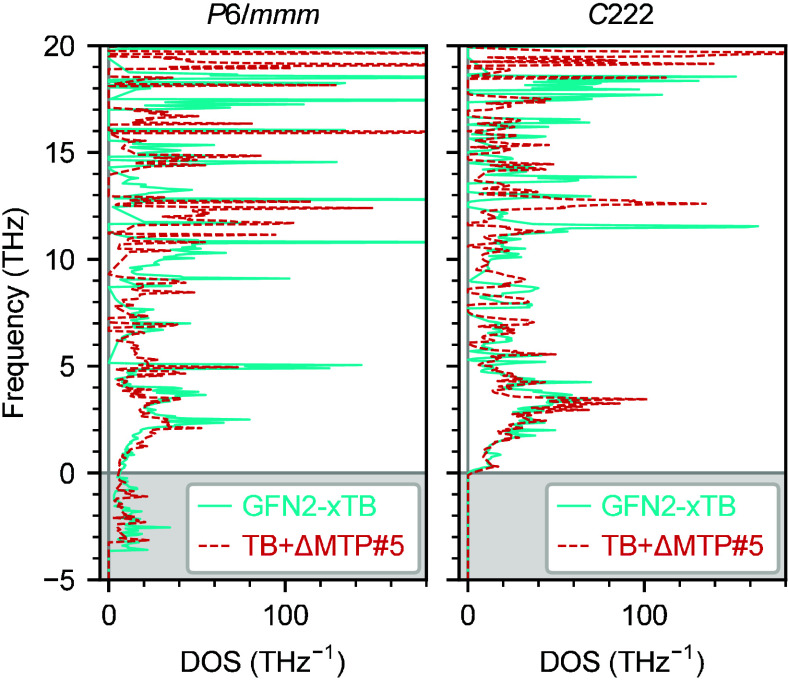
Phonon DOSs of the C_48_H_30_ COF obtained with GFN2-xTB and TB+ΔMTP#5. The negative-frequency region corresponds to imaginary modes.

**Table 5:** Relative Energies Δ*E* (meV/atom) of the *C*222 Structure with Respect to *P*6/*mmm* and Interlayer Distances *d*_layer_ (Å) of the C_48_H_30_ COF

		*d*_node_		*d*_layer_	
	Δ*E*	*P*6/*mmm*	*C*222	*P*6/*mmm*	*C*222
GFN2-xTB	–11.8	12.962	12.766	3.325	3.427
PBE-D4	–10.7	13.032	12.848	3.667	3.678
**TB+ΔMTP#5**	–10.2	13.007	12.891	3.683	3.673

Table 5 also summarizes the obtained node and interlayer distances of the C_48_H_30_ COF. The node-distances, i.e., the distances between the centers of the benzene rings at the end of the *p*-quaterphenyl part, of the *C*222 C_48_H_30_ COF are 12.891 Å and in good agreement with experimentally measured values of 12.9(6) Å^121^ and 13.6(6) Å.^122^ The interlayer distances of the *C*222 C_48_H_30_ COF are 3.673 Å, substantially larger than those of graphite in experiments, 3.35–3.36 Å.^162,163^ The interlayer distances with TB+ΔMTP#5 are substantially larger than the values in GFN2-xTB while in a similar range to PBE-D4, consistent with the intermolecular distances for a benzene–benzene dimer in Figure 7.

Based on the findings of layer offsets in previous atomistic simulations and experiments for general quasi-2D COFs,^164–169^ we also investigated the shearing from the *C*222 structure, but the obtained structure was substantially higher in energy than the unsheared one. Notably, the detailed symmetry of COFs was not discussed in the previous database.^111^ The results imply the possibility for further improvement of COF structures in the existing database, which may eventually improve the prediction of COF properties based on such a database.

Figure 10 shows the local extrapolation grades obtained with ΔMTPs for the C_48_H_30_ COF with the two structures. Like the molecular test data set (Figure 2(c)), the extrapolation grades decrease with increasing training-data set size from #3 toward #5. Particularly for ΔMTP#5, the grades are less than 2 for all the atoms, indicating that the prediction for the present COF should be reliable. It is worth emphasizing that, although the direct energy comparison with the reference CCSD­(T)-level energy for the periodic COF is impractical due to the computational cost, we can still evaluate the transferability of the ΔMTPs with the extrapolation grades as demonstrated.

**Figure 10:**
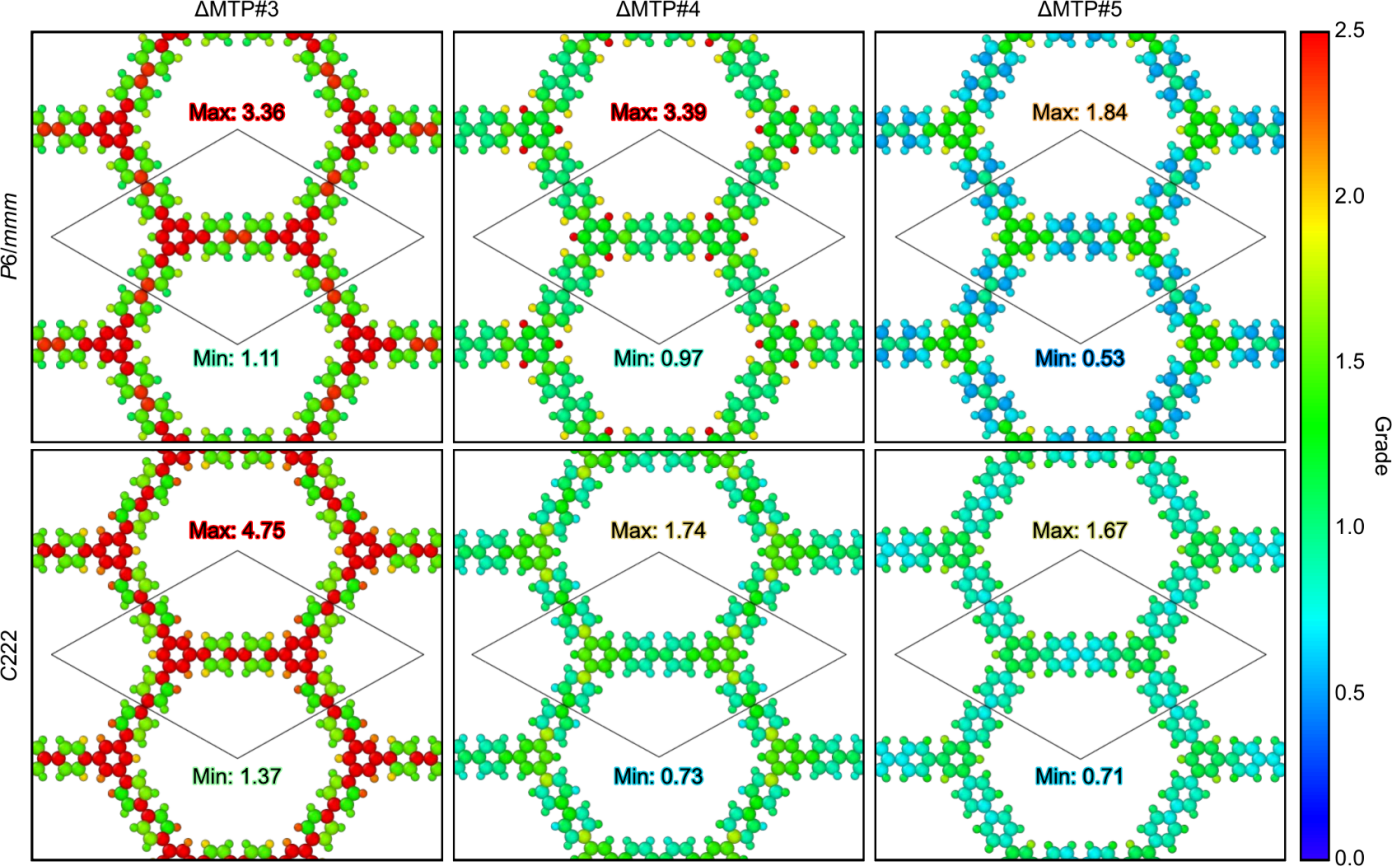
Local extrapolation grades of the C_48_H_30_ COF obtained with ΔMTPs.

Figure 11 shows the interlayer binding energies of the C_48_H_30_ COF with the *C*222 symmetry computed with GFN2-xTB and TB+ΔMTP#5 as a function of interlayer distance. The calculations are performed by modifying the interlayer distance and fixing the intralayer geometry. The reference single-layer energy is obtained using cells with vacuum layer thicknesses larger than 40 Å. TB+ΔMTP#5 shows a longer equilibrium interlayer distance and lower interlayer binding energies in magnitude than GFN2-xTB, consistent with our findings for the benzene–benzene dimer (Figure 7). The binding energy in TB+ΔMTP#5 is 0.055 J/m^2^. This is nearly four times smaller than the interlayer binding energy of graphite in experiments, 0.19–0.37 J/m^2^,^170–173^ which is qualitatively reasonable because the C_48_H_30_ COF has a more sparse in-layer atomic density than graphite and thus shows weaker London dispersion interaction.

**Figure 11:**
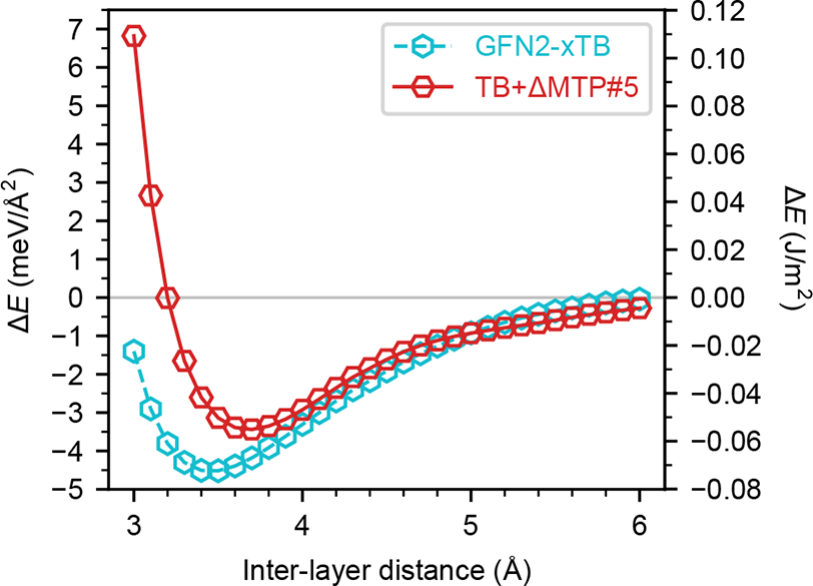
Interlayer binding energies of the C_48_H_30_ COF in the *C*222 symmetry computed with GFN2-xTB and TB+ΔMTP#5 as a function of interlayer distance.

We finally investigate hydrogen absorption on the C_48_H_30_ COF. To compute the H_2_ absorption energies, we put an H_2_ molecule with a random orientation at a random position in the simulation cell and then optimize the structure, while fixing the COF lattice following a previous study.^112^ We do 10 such calculations for each of GFN2-xTB and TB+ΔMTP#5. Figure 12 presents the optimized H_2_ absorption sites with the most negative absorption energies. In both methods, the H_2_ absorption sites with the most negative absorption energies are found near the node position between two adjacent layers. The absorption is weaker in TB+ΔMTP#5 (−0.9 kcal/mol), thus effectively in CCSD­(T), than for GFN2-xTB (−1.1 kcal/mol). It is here worth re-emphasizing that, while TB+ΔMTP#5 exhibits near CCSD­(T) accuracy, its evaluation is computationally even less expensive than DFT and that many such evaluations are affordable. Together with the prediction of phonon DOS, this will be very useful for quantitative predictions in COF research.

**Figure 12:**
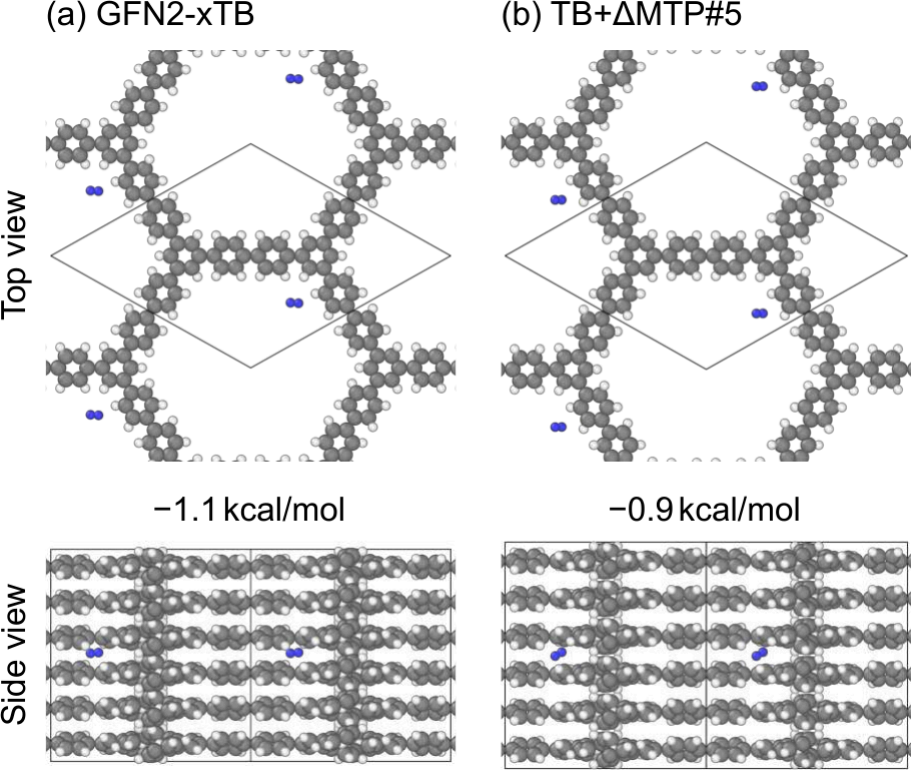
Absorption of an H_2_ molecule (blue) in the C_48_H_30_ COF with the *C*222 symmetry obtained using (a) GFN2-xTB and (b) TB+ΔMTP#5. The corresponding absorption energies are also shown.

## IV Conclusions

We have developed a methodology to train MLIPs with the gold-standard accuracy of quantum chemistry, CCSD­(T), particularly suitable for systems with extended covalent networks and long-range vdW interactions. Based on the Δ-learning approach with a tight-binding baseline, the MLIP can be trained solely with molecular systems, including dispersion-dominated configurations. The thus trained MLIP delivers an RMSE as low as 0.4 meV/atom with respect to the reference PNO-LCCSD­(T)-F12 values and is also seamlessly transferable from molecular to crystalline systems. We have showcased its capabilities by resolving the detailed structural symmetry, the harmonic vibrational frequencies, the interlayer binding energy, and the hydrogen absorption energy of the prototypical COF, C_48_H_30_.

Although the present TB+ΔMTP model is tailored to the C_48_H_30_ COF, the workflow itself is transferable to other systems. Expanding the training set to chemically diverse fragments will broaden the applicability of the potential. The Δ-learning strategy is also straightforward for porting to alternative MLIP architectures. Such extensions will empower high-throughput, CCSD­(T)-quality screening of large libraries of COFs and other vdW-dominated materials, thereby accelerating materials discovery.

## Supplementary Material



## Data Availability

All PNO-LCCSD­(T)-F12 data used for the MLIP training is available at 10.18419/DARUS-5272. Our MTP implementation is available at https://github.com/imw-md/motep.

## Notes

BSSE, basis-set convergence, and the comparison between DFT and RPA for the intermolecular interaction energy of a benzene–benzene dimer with π–π stacking, MTP training procedure, harmonic vibrational frequencies of H_2_ and C_6_H_6_ in various methods, DFT for the C_48_H_30_ COF (PDF)
