# Supplementary material for: Machine-Learning Interatomic Potentials Achieving CCSD(T) Accuracy for Systems with Extended Covalent Networks and van der Waals Interactions
Source: J Chem Theory Comput. 2026 Mar 3;22(6):2739–56. doi: 10.1021/acs.jctc.5c02045 (PMC13019697; doi:10.1021/acs.jctc.5c02045)
Supplement: Supplementary file 1 [file ct5c02045_si_001.pdf]

Supporting Information: Machine-Learning Interatomic Potentials Achieving CCSD(T) Accuracy for Systems with Extended Covalent Networks and van der Waals Interactions

Yuji Ikeda,[1,*] Axel Forslund,[1,2] Pranav Kumar,[1] Yongliang Ou,[1,3]
Jong Hyun Jung,[1] Andreas Köhn,[4] and Blazej Grabowski[1]

[1]*Institute for Materials Science, University of Stuttgart, Pfaffenwaldring 55, 70569 Stuttgart, Germany*
[2]*Department of Materials Science and Engineering, KTH Royal Institute of Technology, SE-100 44 Stockholm, Sweden*
[3]*Department of Materials Science and Engineering, Massachusetts Institute of Technology, 77 Massachusetts Avenue, Cambridge, 02139, MA, USA*
[4]*Institute for Theoretical Chemistry, University of Stuttgart, Pfaffenwaldring 55, 70569 Stuttgart, Germany*

S1. BASIS-SET-SUPERPOSITION ERROR (BSSE)

Intermolecular dispersion interaction energies obtained in quantum-chemical calculations are in general influenced by the basis set superposition error (BSSE); the stability of a multimer is overestimated by the monomer energies evaluated with the basis sets of the whole multimer. It is therefore in principle necessary to compensate this spurious effect in order to evaluate the intermolecular interaction energies. The counterpoise (CP) correction by Boys and Bernardi [S1] is one of the common methods for this compensation.

Figure S1 and Table S1 present the inter-molecular interaction energies of a benzene–benzene dimer with π–π stacking in the S66x8 dataset [S2] obtained with various local correlation methods without and with the CP correction. The heavy-aug-cc-pVTZ basis set is employed. In the calculations with core-electron excitation, the excitation is considered for both the correlation part and the CABS singles correction.

Without the F12 explicit electronic correlation (top row), the impact of the CP correction is substantial and larger in the all-electron treatment (1.1–1.2 kcal/mol) than in the frozen-core approximation (0.6 kcal/mol) at $r_e = 1.00$. In contrast, with the F12 explicit correlation (bottom row), the impact of the CP correction is marginal and less than 0.1 kcal/mol. Thus, the combination of the local correlation methods and the F12 explicit correlation method significantly reduces the BSSE, consistent with the report by Ma and Werner [S4]. The BSSE in these approaches is much smaller than the accuracy that can be achieved by the present MLIP formalism, i.e., 0.1–1 meV/atom and hence essentially negligible for the training of MLIPs. Further, the core-electron treatment does not substantially affect the inter-molecular interaction energies, unlike TAEs (Sec. III A in the main text).

S2. BASIS-SET DEPENDENCE

The correlation-consistent polarized valence basis sets of Dunning [S5], possibly augmented with diffuse functions [S6], are the standard of modern quantum-chemical calculations. However, they were optimized specifically for the frozen-core approximation and for the non-F12 method. Therefore, various extensions have been developed for the all-electron treatment and the F12 method. Namely, the (aug-)cc-pwCV*XZ* basis sets [S7, S8] account for core–core and core–valence correlation effects. The (aug-)cc-pV*XZ*-F12 basis sets [S9, S10] are tuned for the F12 method. The cc-pCV*XZ*-F12 basis sets [S11, S12] consider both, core–core/valence correlation effects and a combination with the F12 method.

Figure S2 and Table S2 present the inter-molecular interaction energies of a benzene–benzene dimer with π–π stacking in the PNO-LCCSD(T)-F12 method together with the various triple-ζ basis sets. The “heavy” basis sets have the augmenting diffuse functions only for non-hydrogen atoms. The calculations with the aug-cc-pVTZ-F12 and cc-pwCVTZ-F12 basis sets failed due to intrinsic linear dependence within the basis sets.

The impact of the all-electron treatment is almost negligible irrespective of the investigated basis sets, including those tuned for the calculations including core–core and core–valence correlation effects, i.e., cc-pwCVTZ and its variants. The impact of the CP correction (Section S1) is also marginal for all the investigated basis sets except those without augmenting diffuse functions and without tuning for the F12 method, i.e., cc-pVTZ and cc-pwCVTZ. Aside from these two basis sets, all the values are similar to those with heavy-aug-cc-pVTZ, employed for the main results in the present study. The use of the heavy-aug-cc-pVTZ basis set is thus justified, as the accuracy that can be achieved by the present MLIP formalism is 0.1–1 meV/atom and the differences from the

* yuji.ikeda@imw.uni-stuttgart.de

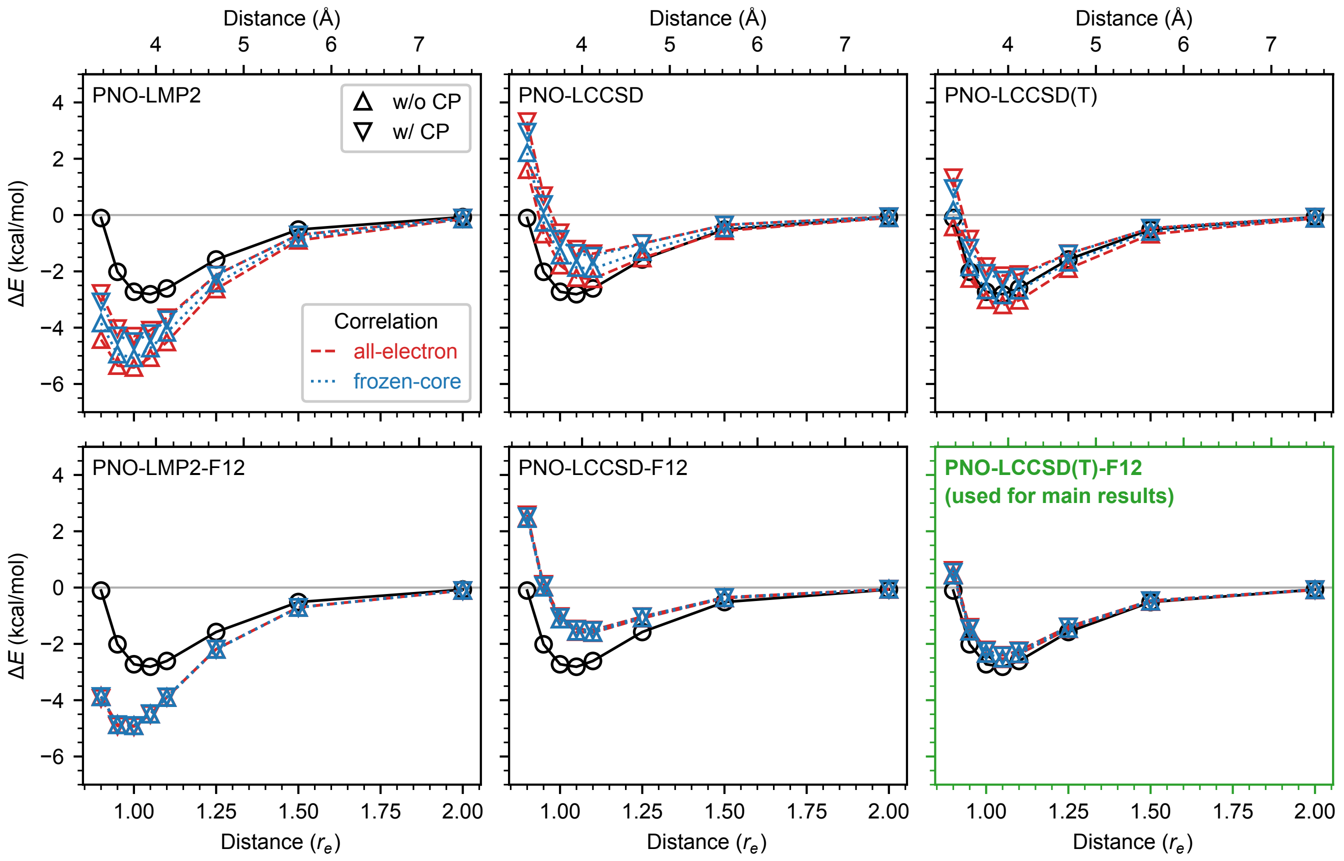

FIG. S1. Inter-molecular interaction energies of a benzene–benzene dimer with π–π stacking obtained with various local correlation methods (kcal/mol). The reference CCSD(F12*)(T)/CBS values [S3] are also shown for comparison in the solid black curves.

other basis sets are hence negligible for the MLIP training. It is also worth noting that, regarding vdW interaction, cc-pVXZ-F12 are not optimized for vdW interaction and indeed reported to be less accurate than aug-cc-pVXZ likely because the former lack higher-angular-momentum diffuse functions [S13–S15], which also supports the use of the standard basis set of Dunning in the present study.

S3. DFT AND RPA

The RPA is often used to validate the accuracy of vdW functionals like optB86b-vdW [S16], rev-vdW-DF2 [S17], SCAN+rVV10 [S18], and r^2SCAN+rVV10 [S19]. We therefore discuss the accuracy of various RPA-based methods available in MOLPRO in this appendix. Specifically, the direct RPA (dRPA) [S20] is the most standard one with the exchange–correlation kernel set to zero. The RPAX2 method [S21] is an extension of the RPA where exchange effects are considered in higher-order particle-hole interactions. The σ-functional method [S22] is based on the adiabatic-connection-fluctuation-dissipation theorem (ACFDT). This method is technically similar to the dRPA and employs a function of Kohn–Sham eigenvalues parametrized based on reference data obtained in experiments or in higher-accuracy computational methods. Note that all-electron treatment seems not available in the σ-functional method with the MOLPRO 2024.1 program.

Figure S3 and Table S3 present the inter-molecular interaction energy of a benzene-benzene dimer with π–π stacking computed with the RPA-based methods on top of the orbitals obtained using the PBE functional [S23]. The results with various DFT functionals are also shown for comparison. The heavy-aug-cc-pVTZ basis set is employed.

Within DFT, the PBE functional fails to capture the attraction between the benzene molecules even qualitatively, in agreement with a previous study [S24]. This well-known deficiency arises from the absence of the vdW interaction, as reported also for layered materials such as graphite [S24–S27]. The LDA appears to reproduce the reference CCSD(F12*)(T)/CBS binding curve semi-quantitatively; however, this apparent agreement is merely fortuitous, stemming from a cancellation of errors between its unphysically long-range exchange interaction and its absence of the vdW interaction [S28, S29]. Consequently, within the DFT framework, a semi-empirical dispersion correction such as the D4 scheme [S30–S32] is necessary.

TABLE S1. Inter-molecular interaction energies of a benzene–benzene dimer with π–π stacking obtained with various local correlation methods (kcal/mol).

			r_e							
Method	Correlation	CP	0.90	0.95	1.00	1.05	1.10	1.25	1.50	2.00
PNO-LMP2	frozen-core	w/o CP	−3.835	−4.942	−5.074	−4.728	−4.170	−2.420	−0.811	−0.142
PNO-LMP2	frozen-core	w/ CP	−3.083	−4.288	−4.492	−4.210	−3.715	−2.137	−0.702	−0.104
PNO-LMP2	all-electron	w/o CP	−4.424	−5.348	−5.417	−5.067	−4.510	−2.630	−0.901	−0.143
PNO-LMP2	all-electron	w/ CP	−2.783	−4.031	−4.302	−4.088	−3.646	−2.129	−0.706	−0.104
PNO-LMP2-F12	frozen-core	w/o CP	−3.885	−4.878	−4.912	−4.498	−3.910	−2.201	−0.710	−0.104
PNO-LMP2-F12	frozen-core	w/ CP	−3.866	−4.863	−4.895	−4.474	−3.878	−2.181	−0.700	−0.102
PNO-LMP2-F12	all-electron	w/o CP	−3.863	−4.867	−4.907	−4.499	−3.900	−2.191	−0.705	−0.108
PNO-LMP2-F12	all-electron	w/ CP	−3.930	−4.916	−4.935	−4.506	−3.894	−2.186	−0.702	−0.102
PNO-LCCSD	frozen-core	w/o CP	+2.212	−0.263	−1.426	−1.866	−1.904	−1.302	−0.463	−0.101
PNO-LCCSD	frozen-core	w/ CP	+2.947	+0.377	−0.858	−1.359	−1.458	−1.021	−0.349	−0.057
PNO-LCCSD	all-electron	w/o CP	+1.609	−0.679	−1.782	−2.225	−2.267	−1.528	−0.561	−0.101
PNO-LCCSD	all-electron	w/ CP	+3.321	+0.689	−0.627	−1.211	−1.371	−1.008	−0.352	−0.056
PNO-LCCSD-F12	frozen-core	w/o CP	+2.447	+0.007	−1.123	−1.554	−1.594	−1.081	−0.369	−0.063
PNO-LCCSD-F12	frozen-core	w/ CP	+2.523	+0.073	−1.060	−1.487	−1.526	−1.037	−0.347	−0.055
PNO-LCCSD-F12	all-electron	w/o CP	+2.481	+0.026	−1.118	−1.568	−1.606	−1.092	−0.378	−0.070
PNO-LCCSD-F12	all-electron	w/ CP	+2.585	+0.117	−1.027	−1.470	−1.506	−1.029	−0.347	−0.055
PNO-LCCSD(T)	frozen-core	w/o CP	+0.172	−1.845	−2.662	−2.840	−2.671	−1.680	−0.585	−0.118
PNO-LCCSD(T)	frozen-core	w/ CP	+0.944	−1.174	−2.066	−2.307	−2.203	−1.385	−0.465	−0.072
PNO-LCCSD(T)	all-electron	w/o CP	−0.425	−2.255	−3.013	−3.193	−3.031	−1.906	−0.683	−0.118
PNO-LCCSD(T)	all-electron	w/ CP	+1.328	−0.852	−1.827	−2.152	−2.110	−1.369	−0.468	−0.071
PNO-LCCSD(T)-F12	frozen-core	w/o CP	+0.436	−1.554	−2.344	−2.516	−2.354	−1.457	−0.489	−0.080
PNO-LCCSD(T)-F12	frozen-core	w/ CP	+0.549	−1.454	−2.250	−2.422	−2.262	−1.399	−0.462	−0.070
PNO-LCCSD(T)-F12	all-electron	w/o CP	+0.465	−1.538	−2.341	−2.532	−2.367	−1.469	−0.499	−0.087
PNO-LCCSD(T)-F12	all-electron	w/ CP	+0.612	−1.408	−2.214	−2.403	−2.241	−1.390	−0.463	−0.070
CCSD(F12*)T/CBS[a]	frozen-core	w/ CP	−0.105	−2.016	−2.725	−2.813	−2.607	−1.578	−0.515	−0.072

[a] Brauer *et al.* [S3]

For the RPA methods, without the CP correction, the results largely depend on the methods and core-electron treatment. For example, in the dRPA without the CP correction, the interaction energy at $r_e = 1.00$ is −3.828 kcal/mol in the frozen-core approximation but −6.343 kcal/mol in all-electron treatment, largely overestimating the interaction energy of the reference CCSD(T)/CBS value in magnitude. With the CP correction, the interaction energies are almost independent of the core-electron treatment; we find the interaction energies of −1.632 kcal/mol and −1.651 kcal/mol in the frozen-core approximation and in all-electron treatment, respectively, which underestimate the reference value by 1.1 kcal/mol in magnitude. The RPAX2 method shows qualitatively the same trend, while the values without the CP correction and with core-electron excitation are much closer to the reference value than dRPA. The insignificance of all-electron treatment in the case with the CP correction implies that its apparent impact is indeed an artifact due to the BSSE and does not actually contribute to the London dispersion interaction. At the same time, the large impact of the CP correction implies that this correction is essential to obtain converged results for the RPA-based methods. Conversely, DFT functionals exhibit negligible impact of the CP correction, implying that the basis-set superposition does not substantially contribute to the description of electron density.

In previous studies, the RPA in conjunction with plane-wave basis sets showed good agreements with experiments for the inter-layer binding energy of graphite [S33], defect formation energies in silicon [S34], the melting temperatures of gold [S35] and silicon [S36], a phase-transition temperature of silica [S37], etc., supporting the predictive power of the RPA for covalent, ionic, metallic, and vdW bonding in high accuracy. With local basis sets, however, the CP correction becomes essential for the RPA, as demonstrated above, making it cumbersome to obtain basis-set converged results. Given also that coupled-cluster theory includes a much broader range of correlation effects than the RPA methods, PNO-LCCSD(T)-F12 is the method of choice for the present study.

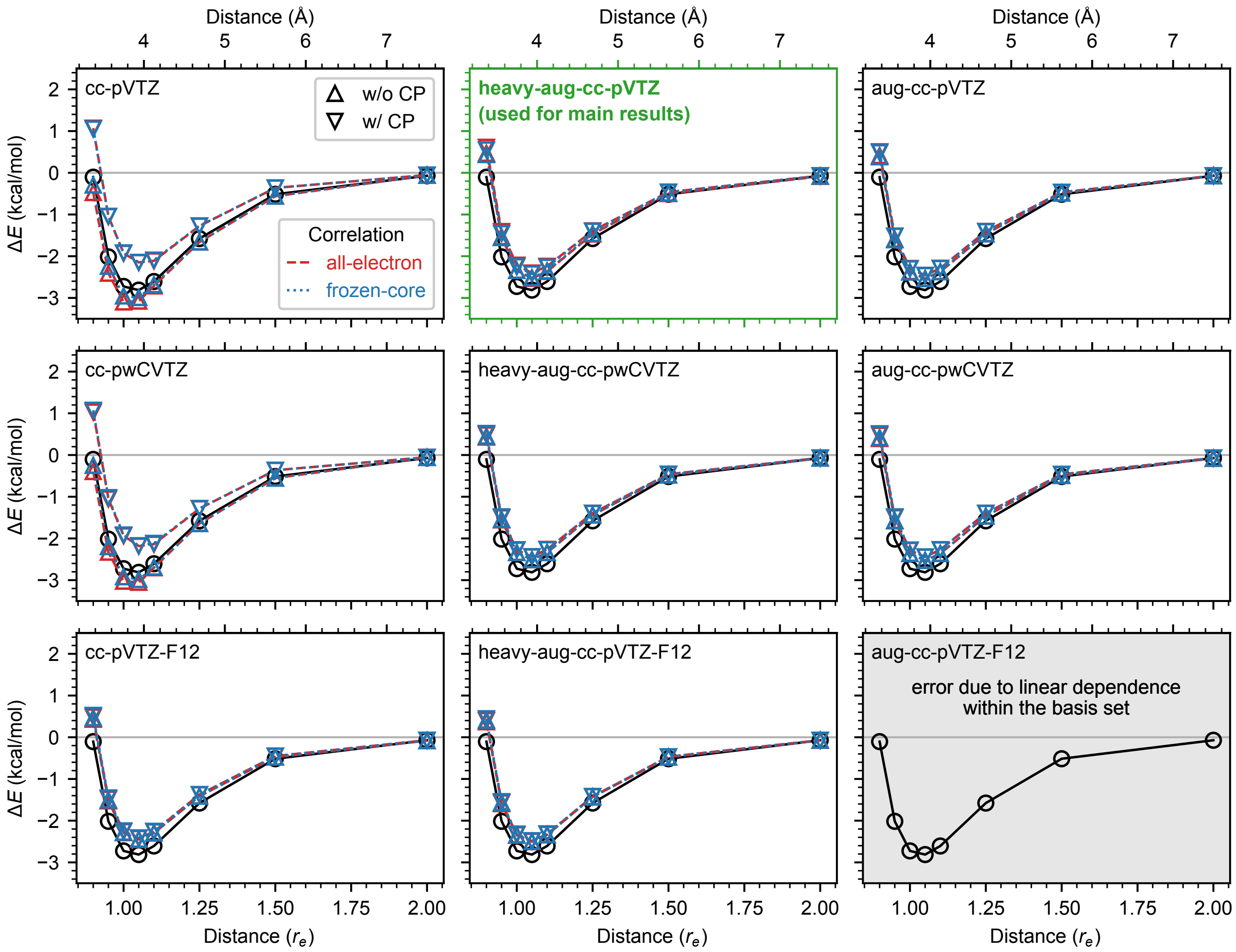

FIG. S2. Basis-set dependence of the inter-molecular interaction energies of a benzene–benzene dimer with π–π stacking obtained with PNO-LCCSD(T)-F12 (kcal/mol). The reference CCSD(F12*)(T)/CBS values [S3] are also shown for comparison in the solid black curves.

TABLE S2. Basis-set dependence of the inter-molecular interaction energies of a benzene–benzene dimer with π–π stacking obtained with PNO-LCCSD(T)-F12 (kcal/mol).

			r_e							
Basis set	Correlation	CP	0.90	0.95	1.00	1.05	1.10	1.25	1.50	2.00
cc-pVTZ	frozen-core	w/o CP	−0.294	−2.241	−2.955	−2.992	−2.685	−1.659	−0.561	−0.059
cc-pVTZ	frozen-core	w/ CP	+1.051	−1.048	−1.919	−2.146	−2.092	−1.271	−0.361	−0.054
cc-pVTZ	all-electron	w/o CP	−0.475	−2.409	−3.098	−3.078	−2.714	−1.660	−0.561	−0.059
cc-pVTZ	all-electron	w/ CP	+1.071	−1.044	−1.928	−2.149	−2.111	−1.277	−0.363	−0.054
heavy-aug-cc-pVTZ	frozen-core	w/o CP	+0.436	−1.554	−2.344	−2.516	−2.354	−1.457	−0.489	−0.080
heavy-aug-cc-pVTZ	frozen-core	w/ CP	+0.549	−1.454	−2.250	−2.422	−2.262	−1.399	−0.462	−0.070
heavy-aug-cc-pVTZ	all-electron	w/o CP	+0.465	−1.538	−2.341	−2.532	−2.367	−1.469	−0.499	−0.087
heavy-aug-cc-pVTZ	all-electron	w/ CP	+0.612	−1.408	−2.214	−2.403	−2.241	−1.390	−0.463	−0.070
aug-cc-pVTZ	frozen-core	w/o CP	+0.383	−1.606	−2.382	−2.539	−2.375	−1.464	−0.489	−0.075
aug-cc-pVTZ	frozen-core	w/ CP	+0.484	−1.518	−2.300	−2.459	−2.291	−1.408	−0.466	−0.070
aug-cc-pVTZ	all-electron	w/o CP	+0.411	−1.593	−2.370	−2.538	−2.371	−1.464	−0.485	−0.070
aug-cc-pVTZ	all-electron	w/ CP	+0.505	−1.516	−2.293	−2.459	−2.287	−1.404	−0.466	−0.070
cc-pwCVTZ	frozen-core	w/o CP	−0.250	−2.197	−2.920	−2.986	−2.689	−1.645	−0.555	−0.059
cc-pwCVTZ	frozen-core	w/ CP	+1.074	−1.020	−1.917	−2.173	−2.117	−1.280	−0.365	−0.054
cc-pwCVTZ	all-electron	w/o CP	−0.397	−2.330	−3.026	−3.053	−2.703	−1.643	−0.553	−0.058
cc-pwCVTZ	all-electron	w/ CP	+1.032	−1.053	−1.935	−2.184	−2.137	−1.285	−0.364	−0.052
heavy-aug-cc-pwCVTZ	frozen-core	w/o CP	+0.452	−1.546	−2.338	−2.509	−2.340	−1.439	−0.480	−0.075
heavy-aug-cc-pwCVTZ	frozen-core	w/ CP	+0.518	−1.480	−2.269	−2.437	−2.268	−1.396	−0.460	−0.069
heavy-aug-cc-pwCVTZ	all-electron	w/o CP	+0.429	−1.557	−2.341	−2.512	−2.339	−1.439	−0.480	−0.076
heavy-aug-cc-pwCVTZ	all-electron	w/ CP	+0.483	−1.502	−2.277	−2.439	−2.261	−1.393	−0.459	−0.069
aug-cc-pwCVTZ	frozen-core	w/o CP	+0.417	−1.569	−2.362	−2.527	−2.362	−1.465	−0.490	−0.079
aug-cc-pwCVTZ	frozen-core	w/ CP	+0.512	−1.480	−2.277	−2.441	−2.271	−1.396	−0.463	−0.070
aug-cc-pwCVTZ	all-electron	w/o CP	+0.394	−1.579	−2.364	−2.530	−2.362	−1.465	−0.491	−0.080
aug-cc-pwCVTZ	all-electron	w/ CP	+0.477	−1.500	−2.284	−2.445	−2.267	−1.393	−0.462	−0.070
cc-pVTZ-F12	frozen-core	w/o CP	+0.466	−1.503	−2.278	−2.446	−2.294	−1.415	−0.477	−0.082
cc-pVTZ-F12	frozen-core	w/ CP	+0.526	−1.454	−2.243	−2.410	−2.254	−1.372	−0.451	−0.070
cc-pVTZ-F12	all-electron	w/o CP	+0.431	−1.527	−2.289	−2.452	−2.297	−1.412	−0.476	−0.082
cc-pVTZ-F12	all-electron	w/ CP	+0.488	−1.481	−2.260	−2.424	−2.265	−1.374	−0.451	−0.070
heavy-aug-cc-pVTZ-F12	frozen-core	w/o CP	+0.410	−1.567	−2.341	−2.501	−2.340	−1.433	−0.477	−0.073
heavy-aug-cc-pVTZ-F12	frozen-core	w/ CP	+0.445	−1.543	−2.320	−2.479	−2.315	−1.418	−0.466	−0.070
heavy-aug-cc-pVTZ-F12	all-electron	w/o CP	+0.370	−1.593	−2.351	−2.503	−2.339	−1.430	−0.477	−0.074
heavy-aug-cc-pVTZ-F12	all-electron	w/ CP	+0.404	−1.570	−2.336	−2.490	−2.324	−1.419	−0.467	−0.071
CCSD(F12*)T/CBS[a]	frozen-core	w/ CP	−0.105	−2.016	−2.725	−2.813	−2.607	−1.578	−0.515	−0.072

[a] Brauer *et al.* [S3]

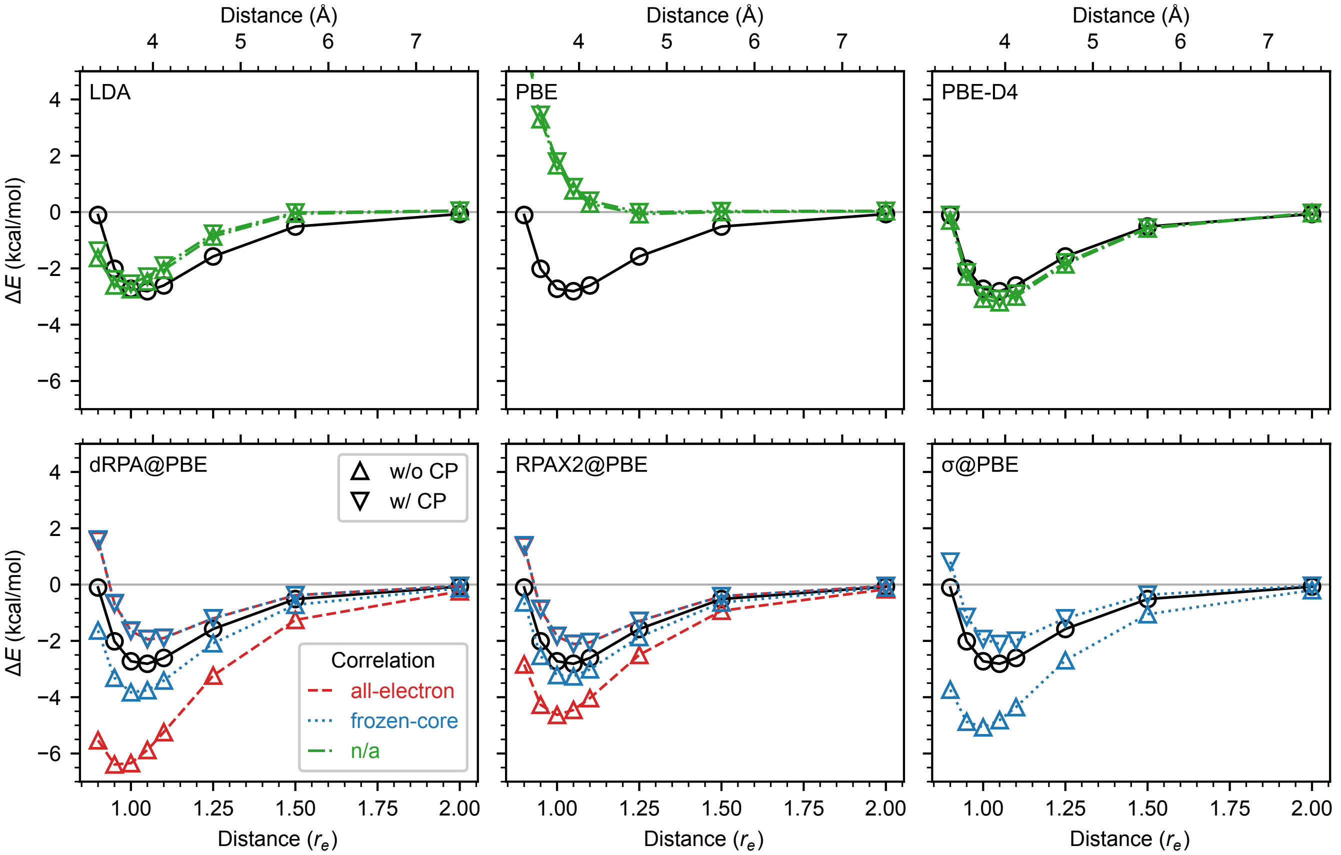

FIG. S3. Intermolecular interaction energies of a benzene–benzene dimer with π–π stacking obtained with various DFT functionals and RPA-based methods (kcal/mol). The reference CCSD(F12*)(T)/CBS values [S3] are also shown for comparison in the solid black curves. Note that all-electron treatment is not available in the σ-functional method likely because the parameters of the σ functionals are optimized only for the frozen-core case.

TABLE S3. Intermolecular interaction energies of a benzene–benzene dimer with π–π stacking obtained with various DFT functionals and RPA-based methods (kcal/mol).

Method	Correlation	CP	r_e							
			0.90	0.95	1.00	1.05	1.10	1.25	1.50	2.00
LDA	n/a	w/o CP	−1.629	−2.607	−2.740	−2.467	−2.053	−0.872	−0.058	+0.038
LDA	n/a	w/ CP	−1.371	−2.390	−2.543	−2.293	−1.898	−0.769	−0.015	+0.050
PBE	n/a	w/o CP	+6.207	+3.300	+1.660	+0.768	+0.298	−0.075	+0.000	+0.033
PBE	n/a	w/ CP	+6.406	+3.452	+1.796	+0.885	+0.404	−0.003	+0.033	+0.042
PBE-D4	n/a	w/o CP	−0.302	−2.293	−3.081	−3.206	−3.002	−1.860	−0.585	−0.036
PBE-D4	n/a	w/ CP	−0.104	−2.141	−2.945	−3.088	−2.896	−1.788	−0.552	−0.027
dRPA@PBE	all-electron	w/o CP	−5.532	−6.390	−6.343	−5.876	−5.241	−3.233	−1.257	−0.240
dRPA@PBE	all-electron	w/ CP	+1.553	−0.688	−1.651	−1.944	−1.900	−1.202	−0.379	−0.046
dRPA@PBE	frozen-core	w/o CP	−1.633	−3.312	−3.828	−3.752	−3.408	−2.086	−0.719	−0.130
dRPA@PBE	frozen-core	w/ CP	+1.601	−0.658	−1.632	−1.930	−1.889	−1.197	−0.376	−0.046
RPAX2@PBE	all-electron	w/o CP	−2.838	−4.271	−4.630	−4.452	−4.033	−2.499	−0.931	−0.168
RPAX2@PBE	all-electron	w/ CP	+1.354	−0.887	−1.839	−2.113	−2.049	−1.295	−0.414	−0.051
RPAX2@PBE	frozen-core	w/o CP	−0.634	−2.534	−3.214	−3.263	−3.011	−1.863	−0.637	−0.107
RPAX2@PBE	frozen-core	w/ CP	+1.396	−0.861	−1.822	−2.102	−2.041	−1.291	−0.413	−0.051
RIRPA@PBE	frozen-core	w/o CP	−3.734	−4.872	−5.071	−4.814	−4.352	−2.691	−1.054	−0.200
RIRPA@PBE	frozen-core	w/ CP	+0.814	−1.151	−1.934	−2.110	−1.993	−1.200	−0.360	−0.041
CCSD(F12*)T/CBS[a]	frozen-core	w/ CP	−0.105	−2.016	−2.725	−2.813	−2.607	−1.578	−0.515	−0.072

[a] Brauer *et al.* [S3]

S4. TRAINING PROCEDURE

An MTP has essentially three types of parameters:

1. `radial_coeffs`: Coefficients for radial functions denoted as $c^{(\beta)}_{\mu,z_i,z_j}$ in Eq. (4) in Podryabinkin *et al.* [S38].
2. `moment_coeffs`: Coefficients for MTP basis functions denoted as ξ_α in Eq. (3) in the main text or in Eq. (2) in Ref. [S38].
3. `species_coeffs`: Reference free-atom energies denoted explicitly as $V_0(z_i)$ in Eq. (3) in the main text.

Among them, `species_coeffs` were fixed to the PNO-LCCSD(T)-F12 values in the present study, and therefore the remaining two types of coefficients were optimized. Figure S4(a) shows the training procedure of the ΔMTPs. Notably, before the main non-linear BFGS fitting, the procedure involves "pre-optimization" of `radial_coeffs` and `moment_coeffs`. Specifically, at the first step, `radial_coeffs` were computed at MTP level 2 in a deterministic manner with linear fitting. The linear fitting for `radial_coeffs` is possible at MTP level 2, because the only MTP basis function is an identity scalar moment tensor, and therefore the MTP reduces to a pair-interaction potential represented by linear combinations of polynomials. (Note that at higher MTP levels the energy depends on `radial_coeffs` non-linearly, and hence the linear fitting is not possible.) At the second step of pre-optimization, `moment_coeffs` were computed deterministically with linear fitting with fixing the already-initialized `radial_coeffs`.

The performance of the pre-optimization process is demonstrated with the RMSEs for the training dataset #5 as given in Figure S4(b), with comparison to the results of the ΔMTPs without the pre-optimization process. The ΔMTPs without the pre-optimization process show one order larger RMSEs than those with the pre-optimization at all the investigated MTP levels. This indicates that the pre-optimization process sets a reasonable initial guess for `radial_coeffs` and `moment_coeffs`, while a much more BFGS steps would be necessary to achieve the same order of RMSEs without the pre-optimization process. This demonstrates that the pre-optimization of the radial and the moment coefficients accelerates the training of MTPs, which is therefore adopted in the present study.

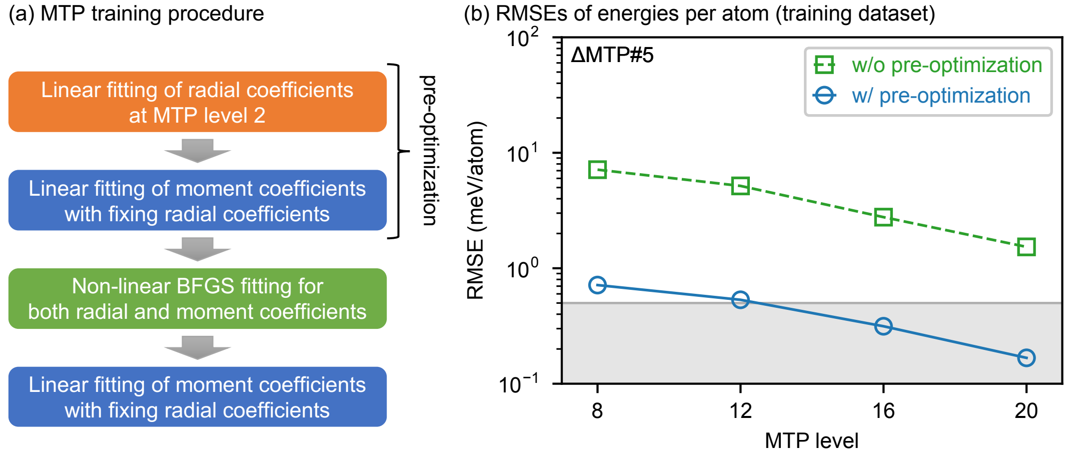

FIG. S4. (a) Training procedures of ΔMTPs. (b) RMSEs for the training dataset #5 without and with the pre-optimization process.

S5. VIBRATIONAL FREQUENCIES

Tables S4 and S5 show the harmonic vibrational frequencies of H_2 and C_6H_6, respectively, computed with GFN2-xTB, various quantum-chemical methods, ANI-1ccx, and TB+ΔMTP#5.

TABLE S4. Harmonic vibrational frequencies of H_2 (cm^{-1}).[a,b]

Method	Σ_g^+
GFN2-xTB	3755
HF	4589
LDA	4184
PBE	4317
PBE-D4	4317
MP2	4528
MP2-F12	4521
CCSD	4411
CCSD-F12	4414
ANI-1ccx	2867
**TB+ΔMTP#5**	4411
Exp.[c]	4401.213

[a] The irreducible representation follows, e.g., Herzberg [S39] and Dresselhaus *et al.* [S40]

[b] The heavy-aug-cc-pVTZ basis set was used for quantum-chemical calculations.

[c] Harmonic vibrational frequencies in Huber and Herzberg [S41].

TABLE S5. Harmonic vibrational frequencies of C_6H_6 (cm^{-1}).[a,b,c]

		1	2	3	4	5	6	7	8	9	10
Method	Correlation	A_{1g}	A_{1g}	A_{2g}	B_{2g}	B_{2g}	E_{2g}	E_{2g}	E_{2g}	E_{2g}	E_{1g}
GFN2-xTB	n/a	1067	3093	1304	658	937	579	3072	1600	1198	882
HF	n/a	1071	3349	1498	768	1129	662	3320	1770	1281	957
LDA	n/a	1017	3110	1317	705	985	602	3088	1619	1150	833
PBE	n/a	995	3123	1332	703	985	599	3097	1590	1159	837
PBE-D4	n/a	995	3123	1332	703	985	599	3097	1590	1159	837
MP2	frozen-core	1011	3232	1369	655	984	606	3205	1633	1192	861
	all-electron	1028	3234	1373	700	1073	607	3193	1640	1188	885
MP2-F12	frozen-core	1014	3233	1377	705	1018	610	3210	1640	1196	867
	all-electron	1016	3228	1380	728	1044	612	3205	1645	1195	867
CCSD	frozen-core	1023	3229	1390	666	1008	617	3200	1666	1203	876
	all-electron	1040	3232	1395	708	1106	618	3186	1672	1199	902
CCSD-F12	frozen-core	1026	3231	1397	712	1034	620	3205	1672	1206	883
	all-electron	1029	3225	1398	723	1063	621	3195	1676	1204	885
CCSD(T)	frozen-core	1002	3202	1369	646	976	606	3173	1630	1186	855
	all-electron	1020	3205	1374	690	1074	608	3160	1637	1182	881
CCSD(T)-F12	frozen-core	1006	3205	1377	692	1003	610	3179	1638	1189	862
	all-electron	1009	3199	1377	705	1033	610	3169	1641	1187	864
ANI-1ccx	n/a	1062	3272	1367	616	1014	621	3230	1693	1210	882
**TB+ΔMTP#5**	n/a	1010	3219	1395	716	1004	606	3172	1629	1188	887
Exp.[d]	n/a	1008	3208	1390	718	1011	613	3191	1639	1192	866

		11	12	13	14	15	16	17	18	19	20
Method	Correlation	A_{2u}	B_{1u}	B_{1u}	B_{2u}	B_{2u}	E_{2u}	E_{2u}	E_{1u}	E_{1u}	E_{1u}
GFN2-xTB	n/a	693	957	3069	1320	1176	368	931	1090	1460	3084
HF	n/a	760	1096	3308	1339	1175	451	1108	1128	1633	3338
LDA	n/a	658	996	3078	1391	1123	396	957	1041	1467	3102
PBE	n/a	665	991	3087	1343	1136	396	957	1034	1465	3113
PBE-D4	n/a	665	991	3087	1343	1136	396	957	1034	1465	3113
MP2	frozen-core	688	1015	3191	1463	1165	399	983	1058	1503	3222
	all-electron	708	1030	3168	1486	1162	408	1033	1063	1513	3221
MP2-F12	frozen-core	690	1026	3200	1455	1167	409	988	1063	1509	3226
	all-electron	691	1034	3195	1459	1167	411	996	1064	1512	3221
CCSD	frozen-core	699	1024	3187	1313	1161	406	1005	1069	1528	3218
	all-electron	720	1040	3162	1342	1159	416	1059	1074	1539	3217
CCSD-F12	frozen-core	700	1036	3194	1317	1163	414	1014	1073	1534	3221
	all-electron	702	1043	3182	1326	1162	414	1025	1074	1536	3214
CCSD(T)	frozen-core	683	1009	3160	1333	1155	395	978	1051	1503	3191
	all-electron	705	1024	3134	1361	1152	405	1033	1056	1513	3190
CCSD(T)-F12	frozen-core	685	1021	3168	1337	1157	403	987	1056	1509	3196
	all-electron	687	1028	3156	1346	1156	403	999	1056	1511	3188
ANI-1ccx	n/a	789	998	3220	1290	1162	390	994	1074	1487	3245
**TB+ΔMTP#5**	n/a	673	1017	3177	1296	1175	407	975	1066	1508	3186
Exp.[d]	n/a	686	1024	3172	1318	1167	407	989	1058	1512	3191

[a] The mode numbers follow Wilson [S42].

[b] The irreducible representations follow, e.g., Mulliken [S43, S44], Tisza [S45], Herzberg [S39], Dresselhaus *et al.* [S40], and Bradley and Cracknell [S46].

[c] The heavy-aug-cc-pVTZ basis set was used for quantum-chemical calculations.

[d] Harmonic vibrational frequencies estimated by Handy *et al.* [S47] based on the fundamental vibrational frequencies of Goodman *et al.* [S48]

S6. DFT FOR THE $C_{48}H_{30}$ COF

For comparison with GFN2-xTB and TB+ΔMTP for the $C_{48}H_{30}$ COF in Sec. III F in the main text, the calculations with a vdW DFT functional, specifically the PBE functional with the D4 correction [S31], were also conducted using the VASP code [S49–S51]. Plane-wave basis sets were employed in conjunction with the plane-wave projector augmented wave (PAW) method [S52]. The plane-wave cutoff energy was set to 520 eV. The reciprocal spaces of the six-layer supercell models, including 468 atoms, were sampled by a Γ-point-only mesh in conjunction with the Gaussian smearing with a width of 0.05 eV. The 2s2p orbitals of C and the 1s orbital of H were treated as valence states. Total energies were minimized until they converged within 1×10^{-7} eV per simulation cell for each self-consistent-field cycle. Structure relaxations were performed until all forces on atoms converged within 5×10^{-3} eV/Å.

The convergences of the energies and the electronic DOSs with respect to k-point-mesh density are tested for the structures optimized with the Γ-point-only meshes. Specifically, $1 \times 1 \times 1$, $1 \times 1 \times 2$, $2 \times 2 \times 1$, and $2 \times 2 \times 2$ meshes were considered, where the first two and the last one numbers correspond to the in-plane and the out-of-plane directions, respectively. The results are summarized in Figure S5.

The energy differences between the Γ-point-only meshes and the $2 \times 2 \times 2$ meshes are less than 0.04 meV/atom for the $P6/mmm$ structure and 0.01 meV/atom for the $C222$ structure, which are one order of magnitude smaller than the fitting error of ΔMTP#5 (0.4 meV/atom, cf. Sec. III A in the main text) and three orders of magnitude smaller than the energy difference between the $P6/mmm$ and the $C222$ structures (10.7 meV/atom, cf. Sec. III F in the main text).

The electronic DOSs with the Γ-point-only meshes also reproduce the characteristic features of the DOSs with the higher k-point-mesh densities, such as the band gaps and the peak positions. The band gaps in PBE-D4 are calculated to be 0.8 eV and 1.5 eV for the $P6/mmm$ and the $C222$ structures, respectively, although we note that band gaps in DFT are typically underestimated.

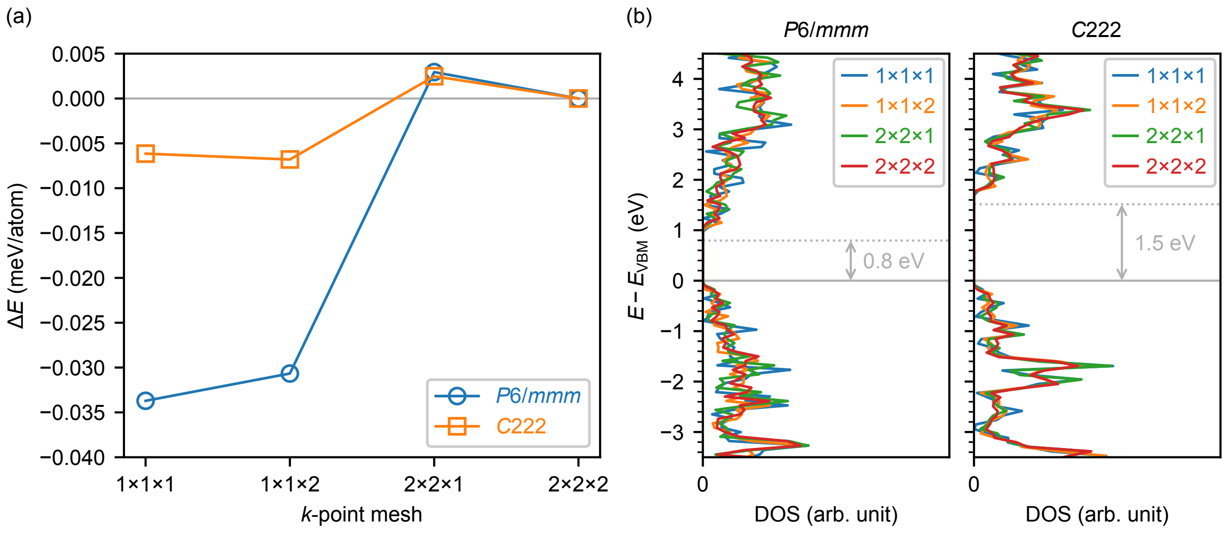

FIG. S5. (a) Energies of the 468-atom supercell models of the $C_{48}H_{30}$ COF with different k-point-mesh densities. For each structure, the energy with the $2 \times 2 \times 2$ k-point mesh is set as the reference. (b) Electronic DOSs of the $C_{48}H_{30}$ COF with different k-point-mesh densities.

[S1] S. Boys and F. Bernardi, The calculation of small molecular interactions by the differences of separate total energies. some procedures with reduced errors, Mol. Phys. **19**, 553 (1970).
[S2] J. Řezáč, K. E. Riley, and P. Hobza, S66: A well-balanced database of benchmark interaction energies relevant to biomolecular structures, J. Chem. Theory Comput. **7**, 2427 (2011); Erratum to "S66: A well-balanced database of benchmark interaction energies relevant to biomolecular structures", J. Chem. Theory Comput. **10**, 1359 (2014).
[S3] B. Brauer, M. K. Kesharwani, S. Kozuch, and J. M. L. Martin, The S66x8 benchmark for noncovalent interactions revisited: explicitly correlated *ab initio* methods and density functional theory, Phys. Chem. Chem. Phys. **18**, 20905 (2016).
[S4] Q. Ma and H.-J. Werner, Explicitly correlated local coupled-cluster methods using pair natural orbitals, WIREs Comput. Mol. Sci. **8**, e1371 (2018).
[S5] T. H. Dunning, Gaussian basis sets for use in correlated molecular calculations. I. the atoms boron through neon and hydrogen, J. Chem. Phys. **90**, 1007 (1989).
[S6] R. A. Kendall, T. H. Dunning, and R. J. Harrison, Electron affinities of the first-row atoms revisited. systematic basis sets and wave functions, J. Chem. Phys. **96**, 6796 (1992).
[S7] K. A. Peterson and T. H. Dunning, Accurate correlation consistent basis sets for molecular core–valence correlation effects: The second row atoms Al–Ar, and the first row atoms B–Ne revisited, J. Chem. Phys. **117**, 10548 (2002).
[S8] N. J. DeYonker, K. A. Peterson, and A. K. Wilson, Systematically convergent correlation consistent basis sets for molecular core–valence correlation effects: The third-row atoms gallium through krypton, J. Phys. Chem. A **111**, 11383 (2007).
[S9] K. A. Peterson, T. B. Adler, and H.-J. Werner, Systematically convergent basis sets for explicitly correlated wavefunctions: The atoms H, He, B–Ne, and Al–Ar, J. Chem. Phys. **128**, 084102 (2008).
[S10] N. Sylvetsky, M. K. Kesharwani, and J. M. L. Martin, The aug-cc-pVnZ-F12 basis set family: Correlation consistent basis sets for explicitly correlated benchmark calculations on anions and noncovalent complexes, J. Chem. Phys. **147**, 134106 (2017).
[S11] J. G. Hill, S. Mazumder, and K. A. Peterson, Correlation consistent basis sets for molecular core-valence effects with explicitly correlated wave functions: The atoms B–Ne and Al–Ar, J. Chem. Phys. **132**, 054108 (2010).
[S12] J. G. Hill and K. A. Peterson, Correlation consistent basis sets for explicitly correlated wavefunctions: valence and core–valence basis sets for Li, Be, Na, and Mg, Phys. Chem. Chem. Phys. **12**, 10460 (2010).
[S13] K. Patkowski, On the accuracy of explicitly correlated coupled-cluster interaction energies — have orbital results been beaten yet?, J. Chem. Phys. **137**, 034103 (2012).
[S14] J. A. Platts, J. G. Hill, K. E. Riley, J. Řezáč, and P. Hobza, Basis set dependence of interaction energies computed using composite post-MP2 methods, J. Chem. Theory Comput. **9**, 330 (2012).
[S15] D. A. Sirianni, L. A. Burns, and C. D. Sherrill, Comparison of explicitly correlated methods for computing high-accuracy benchmark energies for noncovalent interactions, J. Chem. Theory Comput. **13**, 86 (2017).
[S16] J. Klimeš, D. R. Bowler, and A. Michaelides, Van der Waals density functionals applied to solids, Phys. Rev. B **83**, 195131 (2011).
[S17] I. Hamada, van der Waals density functional made accurate, Phys. Rev. B **89**, 121103 (2014).
[S18] H. Peng, Z.-H. Yang, J. P. Perdew, and J. Sun, Versatile van der Waals density functional based on a meta-generalized gradient approximation, Phys. Rev. X **6**, 041005 (2016).
[S19] J. Ning, M. Kothakonda, J. W. Furness, A. D. Kaplan, S. Ehlert, J. G. Brandenburg, J. P. Perdew, and J. Sun, Workhorse minimally empirical dispersion-corrected density functional with tests for weakly bound systems: r^2SCAN+rVV10, Phys. Rev. B **106**, 075422 (2022).
[S20] F. Furche, Molecular tests of the random phase approximation to the exchange-correlation energy functional, Phys. Rev. B **64**, 195120 (2001).
[S21] A. Heßelmann, Random-phase-approximation correlation method including exchange interactions, Phys. Rev. A **85**, 012517 (2012).
[S22] E. Trushin, A. Thierbach, and A. Görling, Toward chemical accuracy at low computational cost: Density-functional theory with σ-functionals for the correlation energy, J. Chem. Phys. **154**, 014104 (2021).
[S23] J. P. Perdew, K. Burke, and M. Ernzerhof, Generalized gradient approximation made simple, Phys. Rev. Lett. **77**, 3865 (1996); Generalized gradient approximation made simple [Phys. Rev. Lett. 77, 3865 (1996)], Phys. Rev. Lett. **78**, 1396 (1997).
[S24] V. Barone, M. Casarin, D. Forrer, M. Pavone, M. Sambi, and A. Vittadini, Role and effective treatment of dispersive forces in materials: Polyethylene and graphite crystals as test cases, J. Comput. Chem. **30**, 934 (2008).
[S25] N. Mounet and N. Marzari, First-principles determination of the structural, vibrational and thermodynamic properties of diamond, graphite, and derivatives, Phys. Rev. B **71**, 205214 (2005).
[S26] J. Park, B. D. Yu, and S. Hong, Ab initio calculations with van der waals corrections: Benzene-benzene intermolecular case and graphite, J. Korean Phys. Soc. **59**, 196 (2011).
[S27] C. R. C. Rêgo, L. N. Oliveira, P. Tereshchuk, and J. L. F. Da Silva, Comparative study of van der waals corrections to the bulk properties of graphite, J. Phys.: Condens. Matter **27**, 415502 (2015).
[S28] J. Harris, Simplified method for calculating the energy of weakly interacting fragments, Phys. Rev. B **31**, 1770 (1985).
[S29] T. Björkman, A. Gulans, A. V. Krasheninnikov, and R. M. Nieminen, Are we van der Waals ready?, J. Phys.: Condens. Matter **24**, 424218 (2012).
[S30] E. Caldeweyher, C. Bannwarth, and S. Grimme, Extension of the D3 dispersion coefficient model, J. Chem. Phys. **147**, 10.1063/1.4993215 (2017).
[S31] E. Caldeweyher, S. Ehlert, A. Hansen, H. Neugebauer, S. Spicher, C. Bannwarth, and S. Grimme, A generally applicable atomic-charge dependent London dispersion correction, J. Chem. Phys. **150**, 154122 (2019).
[S32] E. Caldeweyher, J.-M. Mewes, S. Ehlert, and S. Grimme, Extension and evaluation of the D4 London-dispersion model for periodic

systems, Phys. Chem. Chem. Phys. **22**, 8499 (2020).
[S33] S. Lebègue, J. Harl, T. Gould, J. G. Ángyán, G. Kresse, and J. F. Dobson, Cohesive properties and asymptotics of the dispersion interaction in graphite by the random phase approximation, Phys. Rev. Lett. **105**, 196401 (2010).
[S34] M. Kaltak, J. Klimeš, and G. Kresse, Cubic scaling algorithm for the random phase approximation: Self-interstitials and vacancies in Si, Phys. Rev. B **90**, 054115 (2014).
[S35] B. Grabowski, S. Wippermann, A. Glensk, T. Hickel, and J. Neugebauer, Random phase approximation up to the melting point: Impact of anharmonicity and nonlocal many-body effects on the thermodynamics of Au, Phys. Rev. B **91**, 201103 (2015).
[S36] F. Dorner, Z. Sukurma, C. Dellago, and G. Kresse, Melting Si: Beyond density functional theory, Phys. Rev. Lett. **121**, 195701 (2018).
[S37] A. Forslund, J. H. Jung, Y. Ikeda, and B. Grabowski, Free-energy perturbation in the exchange-correlation space accelerated by machine learning: application to silica polymorphs, npj Comput. Mater. **12**, 14 (2026).
[S38] E. Podryabinkin, K. Garifullin, A. Shapeev, and I. Novikov, MLIP-3: Active learning on atomic environments with moment tensor potentials, J. Chem. Phys. **159**, 084112 (2023).
[S39] G. Herzberg, *Infrared and Raman spectra of polyatomic molecules*, Vol. 2 (van Nostrand, 1946).
[S40] M. Dresselhaus, G. Dresselhaus, and A. Jorio, *Group Theory: Application to the Physics of Condensed Matter* (Springer Berlin Heidelberg, 2007).
[S41] K. P. Huber and G. Herzberg, *Molecular Spectra and Molecular Structure* (Springer, New York, 1979).
[S42] E. B. Wilson, The normal modes and frequencies of vibration of the regular plane hexagon model of the benzene molecule, Phys. Rev. **45**, 706 (1934).
[S43] R. S. Mulliken, Electronic structures of polyatomic molecules and valence. IV. electronic states, quantum theory of the double bond, Phys. Rev. **43**, 279 (1933).
[S44] R. S. Mulliken, Report on notation for the spectra of polyatomic molecules, J. Chem. Phys. **23**, 1997 (1955); Erratum: Report on notation for the spectra of polyatomic molecules, J. Chem. Phys. **24**, 1118 (1956).
[S45] L. Tisza, Zur Deutung der Spektren mehratomiger Moleküle, Z. Phys. **82**, 48 (1933).
[S46] C. J. Bradley and A. P. Cracknell, *The Mathematical Theory of Symmetry in Solids Representation Theory for Point Groups and Space Groups* (Oxford University Press, USA, 2010).
[S47] N. C. Handy, P. E. Maslen, R. D. Amos, J. S. Andrews, C. W. Murray, and G. J. Laming, The harmonic frequencies of benzene, Chem. Phys. Lett. **197**, 506 (1992).
[S48] L. Goodman, A. G. Ozkabak, and S. N. Thakur, A benchmark vibrational potential surface: ground-state benzene, J. Phys. Chem. **95**, 9044 (1991).
[S49] G. Kresse, Ab initio molecular dynamics for liquid metals, J. Non-Cryst. Solids **192-193**, 222 (1995).
[S50] G. Kresse and J. Furthmüller, Efficiency of *ab-initio* total energy calculations for metals and semiconductors using a plane-wave basis set, Comput. Mater. Sci. **6**, 15 (1996).
[S51] G. Kresse and D. Joubert, From ultrasoft pseudopotentials to the projector augmented-wave method, Phys. Rev. B **59**, 1758 (1999).
[S52] P. E. Blöchl, Projector augmented-wave method, Phys. Rev. B **50**, 17953 (1994).
